# Supplementary material for: Association of sociodemographic characteristics with self-perceived access to COVID-19 information and adherence to preventive measures among migrant origin and general populations in Finland: a cross-sectional study
Source: BMJ Open. 2023 Mar 13;13(3):e069192. doi: 10.1136/bmjopen-2022-069192 (PMC10015668; doi:10.1136/bmjopen-2022-069192)
Supplement: Author's manuscript [file bmjopen-2022-069192.draft_revisions.pdf]

BMJ Open is committed to open peer review. As part of this commitment we make the peer review history of every article we publish publicly available.

When an article is published we post the peer reviewers' comments and the authors' responses online. We also post the versions of the paper that were used during peer review. These are the versions that the peer review comments apply to.

The versions of the paper that follow are the versions that were submitted during the peer review process. They are not the versions of record or the final published versions. They should not be cited or distributed as the published version of this manuscript.

BMJ Open is an open access journal and the full, final, typeset and author-corrected version of record of the manuscript is available on our site with no access controls, subscription charges or pay-per-view fees (http://bmjopen.bmj.com).

If you have any questions on BMJ Open's open peer review process please email info.bmjopen@bmj.com

BMJ Open

The association of sociodemographic characteristics with access to Covid-19 information and adherence to preventive measures among migrant origin and general populations in Finland: a cross-sectional study

Journal:	*BMJ Open*
Manuscript ID	bmjopen-2022-069192
Article Type:	Original research
Date Submitted by the Author:	13-Oct-2022
Complete List of Authors:	Skogberg, N. ; National Institute for Health and Welfare, Helsinki, Finland, Welfare<br>Prinkey, Tyler; Finnish Institute for Health and Welfare<br>Lilja, Eero; National Institute for Health and Welfare, Welfare<br>Koponen, Päivikki; National Institute for Health and Welfare, Public Health Solutions<br>Castaneda, Anu; Terveyden ja hyvinvoinnin laitos,
Keywords:	COVID-19, Public health < INFECTIOUS DISEASES, PUBLIC HEALTH

SCHOLARONE™
Manuscripts

I, the Submitting Author has the right to grant and does grant on behalf of all authors of the Work (as defined in the below author licence), an exclusive licence and/or a non-exclusive licence for contributions from authors who are: i) UK Crown employees; ii) where BMJ has agreed a CC-BY licence shall apply, and/or iii) in accordance with the terms applicable for US Federal Government officers or employees acting as part of their official duties; on a worldwide, perpetual, irrevocable, royalty-free basis to BMJ Publishing Group Ltd ("BMJ") its licensees and where the relevant Journal is co-owned by BMJ to the co-owners of the Journal, to publish the Work in this journal and any other BMJ products and to exploit all rights, as set out in our licence.

The Submitting Author accepts and understands that any supply made under these terms is made by BMJ to the Submitting Author unless you are acting as an employee on behalf of your employer or a postgraduate student of an affiliated institution which is paying any applicable article publishing charge ("APC") for Open Access articles. Where the Submitting Author wishes to make the Work available on an Open Access basis (and intends to pay the relevant APC), the terms of reuse of such Open Access shall be governed by a Creative Commons licence – details of these licences and which Creative Commons licence will apply to this Work are set out in our licence referred to above.

Other than as permitted in any relevant BMJ Author's Self Archiving Policies, I confirm this Work has not been accepted for publication elsewhere, is not being considered for publication elsewhere and does not duplicate material already published. I confirm all authors consent to publication of this Work and authorise the granting of this licence.

For peer review only

Title: The association of sociodemographic characteristics with access to Covid-19 information and adherence to preventive measures among migrant origin and general populations in Finland: a cross-sectional study

Natalia Skogberg*[a], Tyler Prinkey[a], Eero Lilja[a], Päivikki Koponen[a], Anu E Castaneda[a]. [The surname of each author is underlined]

*Corresponding author; natalia.skogberg@thl.fi; ORCHID: 0000-0002-8829-4159. Address: Finnish Institute for Health and Welfare, Mannerheimintie 166, P.O. box 27, 00271 Helsinki. Tel. +358 29 524 7916.

[a]Department of Public Health and Welfare, Finnish institute for Health and Welfare, Helsinki, Finland.

word count: 3891

tables: 5

For peer review only - http://bmjopen.bmj.com/site/about/guidelines.xhtml

Abstract

Objectives: This study examines how access to Covid-19 information and adherence to preventive measures varies by sociodemographic characteristics, and whether the associations differ among the migrant origin and the general Finnish population. Additionally, the association of perceived access to information with adherence to preventive measures is examined.

Design: Cross-sectional, population-based random sample.

Background: Equity in access to information is crucial for securing individual wellbeing and successful management of a crisis at population level.

Setting: Persons who have a residence permit in Finland.

Participants: Migrant origin population constitutes of persons born abroad aged 21 to 66 years, who took part in the MigCOVID Survey (n=3 668). Participants in the Health 2017 follow-up survey, representing the general Finnish population consitituted the reference group (n=3 490).

Outcome measures: Self-perceived access to Covid-19 information, adherence to preventive measures.

Results: Self-perceived access to information and adherence to preventive measures was overall high both among the migrant origin and the general population. Perceived adequate access to information was associated with living in Finland for 12 years or longer (OR 1.94, 95%CI 1.05-3.57) and excellent Finnish/Swedish language skills (OR 2.71, 95% CI 1.62-4.53) among the migrant origin population and with higher education (OR 3.56, 95% CI 1.49-8.55 for tertiary and OR 2.87, 95%CI 1.25-6.59 for secondary) among the general population. The association between examined sociodemographic characteristics with adherence to preventive measures varied by study group.

Conclusions: Findings on the association of perceived access to information with language proficiency in official languages highlight the need for rapid multilingual and simple language crisis communications. Findings also suggest that crisis communications and measures designed to influence health behaviours at population level may not be directly transferable if the aim is to influence health behaviours also among ethnically and culturally diverse populations.

key words: migrant, Covid-19, communication, MigCOVID, prevention

For peer review only

Strengths and limitations of the study

- Significant strengths of the current study are its population-based study design, representative sample, and availability of the general population reference group.
- Barriers to participation were reduced by making the survey material available in 18 languages both in electronic and paper-based format, as well as by providing with the option to respond via a telephone interview conducted by multilingual interviewers.
- One limitation is that self-reported access to information does not provide information on the quality of the used sources or whether the information was understood and applied as intended.
- There may be some report bias in the self-reported adherence to preventive measures due to social desirability. It may also be that respondents reported adherence to preventive measures, even though they may have not adhered to them in all situations.

For peer review only

For peer review only - http://bmjopen.bmj.com/site/about/guidelines.xhtml

BACKGROUND

Covid-19 exposed the weakest spots in crisis preparedness, highlighting the evident lack of policies and practices that acknowledge increasing globalization and population cultural diversity[1]. Vulnerabilities of migrant origin populations were reflected in significantly higher incidence of Covid-19 infections, higher risk for hospitalizations, mortality rates, as well as higher economic, social, and psychological impact of the pandemic compared with general populations[2-4]. Information guidance was crucial in management of the pandemic, however crisis communications were challenged by ever-evolving information and changes in official guidelines[5- 6]. From the onset of the pandemic, significant concerns were raised regarding equity in access to information among migrant origin populations. Difficulties in accessing up-to-date and reliable information occurred due to language barriers, lack of trust in the health system, and gaps in health and digital literacy[7-9]. Inequities in access to information may lead to stress, lower awareness, and hence also lower adherence to preventive measures[7,10-11]. In addition to impact on individual-level coping in a crisis, these may negatively impact overall crisis management and hence may jeopardise societal security.

While multilingual communications and community outreach programmes were launched in a number of countries to improve equity in access to information across different population groups[12], practices across countries varied. During the first half year of the pandemic, only half of European countries had information on Covid-19 available in at least one foreign language, and only several countries had information in multiple foreign languages[13]. Significant efforts have been placed to develop multilingual Covid-19 communications also in Finland[14]. A few weeks into the pandemic, the Finnish Institute for Health and Welfare (THL) introduced information on Covid-19 in 16 foreign languages. Language selection was later expanded by several additional languages. It was well acknowledged that simply publishing multilingual information is not sufficient, and particular efforts were taken to ensure effective information dissemination to the target group. Dissemination of national-level Covid-19 communications was implemented by THL's Multilingual and multichannel communications task force in close collaboration with regional health and social welfare professionals, non-governmental organisations, religious community leaders, and other key community representatives[15].

Because multilingual Covid-19 comminications were available with a slight delay, migrant origin populations in Finland initially perceived they did not receive sufficient information, but the situation improved over the course of the pandemic[8]. To date, studies exploring perceived access to information and adherence to preventive measures among migrant origin populations have been generally based on qualitative and non-representative samples[7-9, 16]. The general population reference group is rarely available, which makes contextualisation of the findings challenging. There is also limited information on how access to information and adherence to preventive measures has varied by sociodemographic characteristics, and whether these differed between migrant origin and the general population. Furthermore, there is limited information on how perceived access to information has impacted behaviours in different population groups. This information is needed for evaluation of crisis response measures and for identifying groups at highest risk of vulnerabilities in crisis situations. These all significantly contribute to development of preparedness in future crises. The aim of the current study is to fill this gap in knowledge by using representative population-based data to examine how access to Covid-19 information and adherence to preventive measures varied by sociodemographic characteristics, and whether these differed between the migrant origin and the general Finnish populations. Additionally, the association of access to information with adherence to preventive measures is examined.

For peer review only - http://bmjopen.bmj.com/site/about/guidelines.xhtml

METHODS

The present study is based on the Impact of the Coronavirus on the Wellbeing of the Foreign Born Population (MigCOVID) Survey. The survey was conducted between October 2020 and February 2021, coinciding with the second wave of the Covid-19 pandemic in Finland. A more detailed description of the survey can be found elsewhere[4]. Briefly, the MigCOVID Survey was a 2-year follow-up study to the Survey on Wellbeing among Foreign Born Population[17]. The original stratified random sample was drawn from the Finnish Population Register using the following selection criteria: age 18-64 years, both the person belonging to the sample and their parents were born outside of Finland, and residence in Finland for at least one year. Following exclusion of persons declining further contact during the FinMonik Survey and who no longer lived in Finland, the updated sample of the MigCOVID Survey comprised of 5 269 persons aged 20 to 66 years. In addition to including participants in the MigCOVID Survey, a supplementary sample of 982 persons of Somali origin was drawn using simple random sampling and following the same selection criteria as the original sample. Following exclusion of overcoverage (n=112), the final sample of the MigCOVID Survey constituted of 6 139 persons. Altogether 3 368 persons took part in the MigCOVID Survey, with a participation rate of 60%.

Invitation letters were sent by mail with personal login information to the electronic questionnaire. Data collection was supplemented with mailed paper questionnaires and telephone interviews. Invitation letters, information about the survey, and questionnaires were available in 18 languages. Telephone interviews were carried out by trained multilingual fieldwork personnel in 12 languages. A reference group was formed from a sub-population of the FinHealth 2017 follow-up Study of corresponding age to the participants in the MigCOVID Survey. As the youngest participants in the FinHealth 2017 follow-up Study were 21 years, the reference group of this study constitutes of persons aged 21 to 66 years (n=3 490 participated, participation rate 51%). The FinHealth 2017 Study had a nationally representative, two-stage stratified cluster sample of adults living in Finland[18]. The study was conducted within the same time frame and following a similar protocol to the MigCOVID Survey.

Patient and public involvement in research

This study does not involve patients. The public were not involved in the planning or design of the research. Data collection was implemented by trained multilingual fieldwork personnel. Findings have been disseminated to the public in multiple languages through the webpages of the survey and social media. Additionally, group discussions with representatives of different migrant origin populations have been conducted to disseminate and to hear feedback on the findings.

Variable definitions

Information on date of birth, age, sex, and length of residence in Finland was obtained from the Finnish Population Register. The MigCOVID Survey participants were grouped into six regions of origin based on their country of birth following a slightly modified United Nations area code grouping[19]: Russia or the former Soviet Union (FSU); Estonia; the rest of Europe, North America, and Oceania; the Middle East and North Africa; the rest of Africa; Asia or other (later in the text as Asia). Information on highest completed level of education in Finland or abroad, economic activity, and Finnish/Swedish language skills (official languages of Finland) was gathered during the survey. Categorization of the included sociodemographic characteristics can be found in Table 1.

For peer review only - http://bmjopen.bmj.com/site/about/guidelines.xhtml

Self-perceived access to information was assessed in both studies with the question: Have your received adequate information on how to avoid getting infected with the coronavirus and how to prevent it from spreading? The answer options were: I have not received any information or the information I have received has been completely inadequate; I have received information, but I would have needed more; or I have received adequate information. The first two answer options were grouped.

Adherence to preventive measures was based on the measures recommended by THL at the point of finalization of the questionnaire in September 2020. Participants were asked: Which measures have you taken to avoid getting infected with the coronavirus and prevent it from spreading: I wash my hands more frequently; I use sanitisers more frequently; I take care of hygiene when coughing (e.g. coughing into a disposable tissue, not coughing into hands; I stay at home if I have flu symptoms (e.g. cough, cold symptoms or sore throat); I wear a single use mask or cloth mask during free time (when it is not possible to avoid contact with other people); I keep a safe distance to other people outside of the home; I do not shake hands with the people I meet. The answer options were: Yes, I follow the instruction/ recommendation, and I do not follow the instruction/recommendation. The questions on washing hands, use of sanitisers and coughing hygiene were grouped into a joint variable "improved hand and coughing hygiene". Answers were dichotomized as "yes" (yes to all three) and "no" (no to at least one measure).

Participants were also asked if they have downloaded the Koronavilkku contact tracing app. In the MigCOVID Survey, the answer options were: yes; no, because the app is not available for my phone; no, because I don't know what it is; no, because the app is not available in the languages that I speak; no, for other reasons, what? The answers were dichotomised as yes/no. The open-ended answers were analysed using thematic grouping. In the Health 2017 follow-up Study, the answer options were: yes; no, because I don't have a suitable smartphone; no for other reasons. These were also dichotomized as yes or no.

Statistical analyses

The differences between groups were tested using logistic regression. P-value based on Satterthwaite-adjusted F-value, as well as odds ratio and its 95% confidence interval were used to assess the differences. The model-adjusted proportions were calculated with predictive margins[20]. The stratification of the sample was accounted for in all analyses and finite population correction was applied[21]. The data were weighted using inverse probability weights (IPWs) to reduce the non-response bias and to take varying sampling probabilities into account[22]. The calculation of the IPWs is described elsewhere[4, 18]. All analyses were carried out with SAS 9.4 and SUDAAN 11.0.3 statistical software.

For peer review only

RESULTS

Descriptive characteristics of the study participants are presented in Table 1. The proportion of men was lowest among persons born in Russia/FSU and highest among persons born in the Middle East and North Africa. Persons born in Africa (excl. North Africa) were the youngest. The proportion of those with at least tertiary education was lowest among persons born in Estonia and highest among persons born in the rest of Europe, North America and Oceania. Employment was lowest among persons born in the Middle East and North Africa. Persons born in Russia/FSU had lived in Finland the longest and had the highest Finnish/Swedish language proficiency.

[Table 1 about here]

Perceived access to information and adherence to preventive measures among the study participants is presented in Table 2. Migrant origin population reported receiving fully adequate Covid-19 information less frequently than the general population. Perceived access to information was lowest among persons born in Europe (excl. Russia/FSU, Estonia), North America and Oceania, the Middle East and North Africa, and Asia.

[Table 2 about here]

Persons born in Russia/FSU and Estonia reported lower, while persons born in the Middle East and Africa reported higher adherence to preventive measures compared with the general population. Migrant origin population reported downloading the Koronavilkku contact tracing app less frequently than the general population. The most common reasons mentioned for not downloading the app were not knowing about it; the app was unnecessary; not wanting to; the app would just increase stress; mistrust towards the authorities; technical difficulties; and that the app consumes too much phone memory. (results not shown)

The association of sociodemographic characteristics with self-perceived access to information and adherence to Covid-19 preventive measures among the general population is presented in Table 3. Higher education was positively associated with self-perceived sufficient access to information. Women were more likely than men to report adhering to all other preventive measures except for keeping a safety distance to others. Older age was positively associated with improved hand hygiene and use of a face mask. Additionally, persons in the oldest age group (50-66 years) were more likely to keep a safety distance to others but less likely to download the contact tracing app compared with the youngest (21-34 years) age group. Persons with tertiary education were more likely to report the use of a face mask than persons with basic education. Higher education and employment were also associated with downloading the contact tracing app.

[Table 3 about here]

For peer review only - http://bmjopen.bmj.com/site/about/guidelines.xhtml

Table 1 Descriptive characteristics of the study participants

	General population	Born abroad total	Russia or former Soviet Union	Estonia	Rest of Europe, North America, Oceania	Middle East and North Africa	Rest of Africa	Asia
	n=6543	n=3456	n=1115	n=300	n=712	n=436	n=302	n=746
	N (%)	N (%)	N (%)	N (%)	N (%)	N (%)	N (%)	N (%)
**Men**	1604 (50.5)	1614 (51.6)	380 (39.0)	110 (46.5)	399 (64.7)	286 (68.8)	174 (54.9)	265 (42.7)
**Age**								
21–34 years	533 (29.3)	1090 (34.7)	251 (24.5)	56 (25.0)	179 (32.5)	178 (49.7)	156 (45.7)	270 (38.1)
35–49 years	1142 (33.6)	1509 (41.7)	412 (38.0)	119 (39.7)	339 (48.1)	178 (36.2)	122 (46.9)	339 (43.0)
50–66 years	1815 (37)	1012 (23.6)	452 (37.5)	125 (35.3)	194 (19.4)	80 (14.1)	24 (7.4)	137 (18.9)
**Education**								
Basic education or less	273 (6.5)	380 (13.2)	57 (6.0)	32 (16.0)	55 (11.8)	86 (16.8)	56 (21.0)	94 (14.2)
Secondary education	1764 (49.7)	1276 (46.1)	435 (56.6)	147 (59.2)	210 (33.5)	160 (50.8)	117 (42.4)	207 (36.2)
Tertiary education or higher	1412 (43.8)	1888 (40.8)	609 (37.4)	113 (24.8)	440 (54.6)	174 (32.4)	119 (36.6)	433 (49.6)
**Economic activity**								
Employed full-time/part-time	2404 (70.7)	2299 (65.7)	699 (64.8)	218 (70.9)	504 (72.5)	197 (51.6)	172 (59.9)	509 (69.7)
Unemployed, student or other	1034 (29.3)	1229 (34.3)	396 (35.2)	77 (29.1)	195 (27.5)	225 (48.4)	119 (40.1)	217 (30.3)
**Length of residence in Finland**								
3–6.99 years	N/A	793 (17.9)	163 (6.7)	32 (6.5)	134 (22.6)	183 (31.8)	46 (14.3)	235 (24.0)
7–11.99 years	N/A	1020 (28.7)	273 (18.9)	124 (43.4)	200 (28.2)	116 (30.4)	58 (21.3)	249 (31.6)
12+ years	N/A	1798 (53.4)	679 (74.3)	144 (50.1)	378 (49.2)	137 (37.9)	198 (64.3)	262 (44.3)
**Excellent Finnish/Swedish language proficiency**	N/A	1364 (35.4)	480 (43.2)	182 (52.1)	273 (32.0)	122 (29.1)	163 (50.3)	144 (17.8)

For peer review only - http://bmjopen.bmj.com/site/about/guidelines.xhtml

For peer review only

Table 2 Perceived access to information and self-reported adherence to preventive measures among the study population[1]

	General population n=6543 % (95%CI)	Born abroad total n=3456 % (95%CI)	Russia or former Soviet Union n=1115 % (95%CI)	Estonia n=300 % (95%CI)	Rest of Europe, North America, Oceania n=712 % (95%CI)	Middle East and North Africa n=436 % (95%CI)	Rest of Africa n=302 % (95%CI)	Asia n=746 % (95%CI)
**Access to information**								
Received adequate information	97.1 (95.7–98.0)	**93.5 (92.0–94.8)*** **	96.1 (94.3–97.4)	97.4 (94.0–98.9)	**91.4 (87.0–94.5)*** **	**91.8 (86.6–95.1)** **	93.9 (88.8–96.7)	**91.4 (86.8–94.5)*** **
Received, but would have needed more	2.5 (1.6–3.9)	**5.3 (4.2–6.7)*** **	2.8 (1.8–4.3)	1.9 (0.7–5.5)	**6.2 (3.8–9.9)** **	**7.8 (4.6–13.0)** **	5.4 (2.7–10.6)	**7.5 (4.8–11.6)*** **
No information/completely inadequate	0.4 (0.2–0.8)	**1.1 (0.6–2.0)*** **	**1.1 (0.5–2.4)* **	0.6 (0.2–2.4)	**2.4 (0.9–6.2)** **	0.4 (0.1–1.1)	0.7 (0.3–1.9)	1.1 (0.2–5.6)
**Following preventive measures**								
Staying home with flu symptoms	96.1 (94.6–97.2)	96.4 (95.1–97.4)	94.2 (91.4–96.1)	92.1 (85.2–95.9)	96.4 (92.8–98.2)	**99.2 (98.1–99.7)** **	**99.0 (97.6–99.6)** **	98.2 (95.2–99.3)
Improved hand and coughing hygiene	92.2 (90.3–93.7)	92.8 (91.0–94.2)	**85.3 (81.2–88.7)*** **	**84.4 (75.7–90.4)* **	**96.3 (92.7–98.1)* **	95.7 (91.4–97.9)	**98.7 (96.9–99.5)*** **	96.2 (92.4–98.1)
Use of face mask	81.9 (79.6–83.9)	81.5 (79.0–83.7)	**69.5 (64.1–74.4)*** **	**65.0 (55.5–73.5)*** **	84.2 (78.5–88.5)	**88.9 (83.5–92.7)* **	**93.7 (90.4–96.0)*** **	**89.2 (84.1–92.9)*** **
Keeping a safety distance	94.6 (93.0–95.8)	93.5 (91.6–95.0)	92.0 (88.5–94.6)	**87.1 (78.1–92.8)** **	92.6 (86.6–96.0)	**96.8 (93.0–98.6)**	**97.3 (95.3–98.5)* **	94.9 (90.2–97.4)
Avoiding shaking hands with others	98.8 (98.3–99.2)	**93.2 (91.5–94.6)*** **	**89.7 (86.2–92.3)*** **	**84.7 (75.5–90.9)*** **	**95.8 (92.5–97.7)*** **	**95.4 (92.2–97.4)*** **	**95.7 (92.5–97.6)*** **	**96.2 (92.0–98.3)** **
Downloaded Koronavilkku contact tracing app	62.4 (60.1–64.7)	**44.1 (41.2–47.2)*** **	**33.5 (28.4–39.0)*** **	**23.1 (16.6–31.2)*** **	**53.5 (46.6–60.3)* **	**51.4 (43.7–59.1)** **	**50.8 (40.2–61.4)* **	**52.8 (46.0–59.4)** **

[1]Adjusted for age and sex

*p<0.05; **p<0.01; ***p<0.001, in comparison with the general population

For peer review only - http://bmjopen.bmj.com/site/about/guidelines.xhtml

Table 3 The association of sociodemographic characteristics with self-perceived adequate access to information and adherence to Covid-19 related preventive measures among the general population

	Perceived access to information	Staying home with flu symptoms	Improved hand and coughing hygiene	Use of face mask	Keeping a safety distance to others	Avoiding shaking hands with others	Downloaded Koronavilkku app
	OR (95%CI)	OR (95%CI)	OR (95%CI)	OR (95%CI)	OR (95%CI)	OR (95%CI)	OR (95%CI)
**Sex**[1]							
Men	1.00	1.00	1.00	1.00	1.00	1.00	1.00
Women	0.75 (0.36–1.55)	**2.53 (1.14–5.59)***	**2.43 (1.58–3.74)*****	**3.30 (2.49–4.38)*****	1.17 (0.68–2.02)	**2.46 (1.16–5.19)***	**1.64 (1.36–1.98)*****
**Age**[2]							
21–34 years	1.00	1.00	1.00	1.00	1.00	1.00	1.00
35–49 years	1.51 (0.59–3.89)	1.35 (0.61–3.00)	**1.92 (1.11–3.32)***	**1.50 (1.03–2.21)***	1.24 (0.65–2.39)	2.17 (0.90–5.25)	0.79 (0.58–1.07)
50–66 years	2.08 (0.80–5.36)	2.21 (0.99–4.91)	**2.08 (1.23–3.52)****	**2.05 (1.41–2.97)*****	**2.46 (1.27–4.79)****	1.96 (0.86–4.45)	**0.61 (0.45–0.81)*****
**Education**[3]							
Basic education or less	1.00	1.00	1.00	1.00	1.00	1.00	1.00
Secondary education	**2.87 (1.25–6.59)***	1.13 (0.48–2.69)	1.12 (0.37–3.36)	1.28 (0.88–1.87)	0.94 (0.43–2.04)	1.12 (0.37–3.36)	**2.13 (1.58–2.87)*****
Tertiary education or higher	**3.56 (1.49–8.55)****	1.51 (0.61–3.75)	1.19 (0.37–3.86)	**2.20 (1.41–3.44)*****	1.10 (0.49–2.47)	1.19 (0.37–3.86)	**4.48 (3.22–6.24)*****
**Economic activity**[3]							
Unemployed, student or other	1.00	1.00	1.00	1.00	1.00	1.00	1.00
Employed full-time/part-time	0.88 (0.37–2.09)	1.81 (0.86–3.83)	0.89 (0.40–1.96)	0.93 (0.66–1.30)	0.80 (0.40–1.59)	0.89 (0.40–1.96)	**0.63 (0.50–0.79)*****

[1]Adjusted for age
[2]Adjusted for sex
[3]Adjusted for age and sex
*p<0.05; **p<0.01; ***p<0.001

For peer review only

The association of sociodemographic characteristics with self-perceived access to information and adherence to Covid-19 preventive measures among the migrant origin population is presented in Table 4. Longer length of residence and excellent Finnish/Swedish skills were associated with perceived sufficient information on Covid-19. Women were generally more likely to adhere to preventive measures than men. Older age was associated with a lower likelihood of reporting staying at home with flu symptoms and downloading the Koronavilkku contact tracing app, while tertiary education was associated with a greater likelihood of downloading the Koronavilkku app. Secondary education was associated with being less likely, whereas excellent Finnish/Swedish skills were associated with being more likely to avoid shaking hands with others. Those who have lived in Finland for 7-12 years were less likely to use a face mask than those who have lived in Finland for a shorter period.

[Table 4 about here]

The association of self-perceived access to information with adherence to preventive measures is presented in Table 5. When the migrant origin population was examined as one group, the association was statistically significant for keeping a safety distance to others only. When examined by region of origin, statistically significant differences emerged in the population born in Africa (excl. North Africa) in case of all preventive measures except for downloading the Koronavilkku app. Because of widened conficence intervals in the subgroup analyses, interaction analyses were run to examine whether the associations for this group really differed from other regional groups. The p-values for the differences of Africa (excl. North Africa) group vs. other groups combined were p=0.101 for staying at home with flu symptoms; p=0.018 for following improved hand and coughing hygiene; p=0.074 for using a face mask; p=0.255 for keeping a safety distance to others; p=0.034 for avoiding shaking hands with others; and p=0.504 for downloading the Koronavilkku app. Based on these, it appears that the association of perceived access to information and following preventive measures was different for this regional group.

[Table 5 about here]

For peer review only

Table 4 The association of sociodemographic characteristics with self-perceived adequate access to information, adherence to Covid-19 related preventive measures, and Covid-19 infection among migrant origin persons

	Perceived access to information	Staying home with flu symptoms	Improved hand and coughing hygiene	Use of face mask	Keeping a safety distance to others	Avoiding shaking hands with others	Downloaded Koronavilkku app
	OR (95%CI)	OR (95%CI)	OR (95%CI)	OR (95%CI)	OR (95%CI)	OR (95%CI)	OR (95%CI)
**Sex**[1]							
Men	1.00	1.00	1.00	1.00	1.00	1.00	1.00
Women	1.22 (0.79–1.89)	1.07 (0.60–1.93)	**2.10 (1.33–3.31)****	**1.70 (1.23–2.33)****	**2.12 (1.26–3.56)****	**2.41 (1.48–3.92)*****	1.08 (0.84–1.39)
**Age**[2]							
21-34 years	1.00	1.00	1.00	1.00	1.00	1.00	1.00
35-49 years	0.94 (0.57–1.56)	**0.25 (0.14–0.47)*****	1.01 (0.58–1.76)	1.10 (0.76–1.59)	1.36 (0.73–2.52)	1.06 (0.62–1.82)	**0.61 (0.46–0.82)****
50-66 years	1.39 (0.73–2.66)	**0.24 (0.12–0.48)*****	0.93 (0.51–1.69)	1.30 (0.86–1.97)	2.09 (0.99–4.40)	1.29 (0.68–2.43)	**0.35 (0.25–0.49)*****
**Education**[3]							
Basic education or less	1.00	1.00	1.00	1.00	1.00	1.00	1.00
Secondary education	0.99 (0.50–1.94)	0.58 (0.14–2.40)	0.57 (0.22–1.46)	0.73 (0.42–1.25)	0.87 (0.36–2.10)	**0.38 (0.15–0.95)***	0.94 (0.63–1.41)
Tertiary education or higher	0.66 (0.34–1.27)	0.82 (0.19–3.56)	1.25 (0.49–3.20)	1.08 (0.62–1.88)	0.86 (0.36–2.07)	1.09 (0.42–2.86)	**1.72 (1.15–2.58)****
**Economic activity**[3]							
Unemployed, student or other	1.00	1.00	1.00	1.00	1.00	1.00	1.00
Employed full-time/part-time	0.69 (0.43–1.13)	1.18 (0.63–2.24)	1.21 (0.76–1.94)	1.11 (0.79–1.56)	1.58 (0.87–2.87)	0.87 (0.54–1.38)	0.92 (0.71–1.20)
**Length of residence in Finland**[3]							
3-6.99 years	1.00	1.00	1.00	1.00	1.00	1.00	1.00
7-11.99 years	1.15 (0.63–2.11)	0.93 (0.30–2.84)	0.50 (0.23–1.09)	**0.45 (0.27–0.75)****	1.21 (0.52–2.82)	0.68 (0.34–1.38)	0.92 (0.71–1.20)
12+ years	**1.94 (1.05–3.57)***	0.85 (0.31–2.30)	0.50 (0.25–1.00)	0.62 (0.38–1.01)	1.35 (0.64–2.84)	1.05 (0.56–1.97)	0.92 (0.71–1.20)
**Finnish/Swedish proficiency**[3]							
Intermediate/beginner or less	1.00	1.00	1.00	1.00	1.00	1.00	1.00
Excellent	**2.71 (1.62–4.53)*****	0.98 (0.51–1.86)	0.83 (0.52–1.30)	1.04 (0.76–1.44)	1.28 (0.72–2.26)	**1.73 (1.06–2.84)***	1.13 (0.87–1.47)

[1]Adjusted for age
[2]Adjusted for sex
[3]Adjusted for age and sex
*p<0.05; **p<0.01; ***p<0.001

For peer review only

Table 5 The association of adequate self-perceived access to information with following preventive measures by region of origin[1]

	Staying home with flu symptoms	Following improved hand and coughing hygiene	Using a face mask	Keeping a safety distance to others	Avoiding shaking hands with others	Downloading the Koronavilkku app
	OR (95%CI)	OR (95%CI)	OR (95%CI)	OR (95%CI)	OR (95%CI)	OR (95%CI)
General population	0.58 (0.08–4.31)	0.72 (0.23–2.24)	0.95 (0.46–1.97)	0.99 (0.27–3.59)	1.20 (0.16–9.00)	1.78 (0.90–3.52)
Born abroad total	1.58 (0.60–4.16)	0.73 (0.37–1.43)	1.49 (0.88–2.53)	**2.54 (1.15–5.61)***	1.53 (0.71–3.33)	1.03 (0.63–1.69)
Russia or former Soviet Union	1.01 (0.27–3.74)	1.25 (0.46–3.39)	2.87 (1.35–6.11)	2.51 (0.80–7.90)	1.57 (0.46–5.37)	2.05 (0.87–4.81)
Estonia	3.19 (0.44–23.28)	0.25 (0.04–1.77)	0.46 (0.08–2.62)	4.56 (0.56–37.26)	1.01 (0.15–6.95)	1.18 (0.22–6.37)
Rest of Europe, North America and Oceania	2.86 (0.46–17.83)	0.74 (0.12–4.43)	1.94 (0.57–6.65)	4.22 (0.87–20.40)	**6.36 (1.42–28.51)***	1.38 (0.49–3.85)
Middle East and North Africa	0.54 (0.09–3.41)	1.46 (0.15–14.60)	1.98 (0.56–6.97)	0.61 (0.10–3.77)	0.87 (0.17–4.44)	1.39 (0.45–4.29)
Rest of Africa	**8.29 (1.76–39.01)****	**15.06 (1.19–190.10)***	**6.13 (1.73–21.76)****	**6.63 (1.70–25.86)****	**15.50 (3.44–69.76)*****	0.55 (0.13–2.27)
Asia	0.40 (0.04–3.93)	0.40 (0.09–1.82)	1.56 (0.36–6.71)	2.49 (0.30–20.43)	0.13 (0.01–1.18)	1.08 (0.37–3.14)

[1]Adjusted for age and sex; the reference group are those who percieved they did not receive fully adequate information on COVID-19 and related restrictive measures.

*p<0.05; **p<0.01; ***p<0.001

For peer review only - http://bmjopen.bmj.com/site/about/guidelines.xhtml

DISCUSSION

Perceived access to information and self-reported adherence to preventive measures were high both in the migrant origin and the general population. In the general population, higher education, whereas in the migrant origin population, longer length of residence and excellent Finnish/Swedish language skills were associated with higher perceived access to information. In the general population, being a woman, older age, and higher education were positively associated with adherence to preventive measures. While women reported higher adherence to preventive measures also in the migrant origin population, the association of other sociodemographic characteristics was less consistent than in the general population. Self-perceived sufficient access to information was associated with keeping a safety distance to others in the migrant origin population. In the analyses by region of origin, perceived access to information was associated with adherence to all preventive measures except for downloading the Koronavilkku contact tracing app among persons born in Africa (excl. North Africa).

Despite overall high perceived access to information, migrant origin populations reported they would have needed more information on Covid-19 more frequently than the general population. Furthermore, some regional group differences were observed, which may be attributable to the observed lower Finnish/Swedish language proficiency and shorter length of residence in some groups. Findings on the association of proficiency in Finnish/Swedish with access to information are consistent with findings of a qualitative study conducted among Russian, Arabic, and Somali-speaking populations at the start of the Covid-19 pandemic in Finland[8]. In the current study, perceived access to information was poorer in those who spoke Finnish/Swedish at intermediate level. This may reflect the complexity of crisis communications. The observed association between length of residence with perceived access to information may be related to higher likelihood of attaining better Finnish/Swedish language proficiency with longer length of residence in Finland. It may also reflect accumulation of better general knowledge on where to source relevant information. The observed association of higher education with higher perceived access to information among the general population in this study is consistent with previous studies conducted among general populations[23]. Persons with higher education may be more skilled in seeking health-related information and may have better health literacy[24].

Self-reported adherence to preventive measures was overall high both among the general and migrant origin population in the current study, which is consistent with findings from Norway[16] and Switzerland[25], but contradictory with findings from France[26]. Partially contradictory findings may be attributable to the fact that the study in France focused specifically on a disadvantaged migrant origin group from one specific region of origin, whereas the other studies included more heterogenous populations. In the current study, some differences in adherence to preventive measures were observed by region of origin. Lower adherence to preventive measures among persons born in Russia/FSU and Estonia compared with other groups may partially reflect behavioural patterns and official recommendations in the country of origin of participants. In Russia and Estonia, adherence to preventive measures has been reportedly influence by the authorities' ambivalent and dismissive attitudes and the citisens' perceived lack of support[27-28]. In contrast, persons born in the Middle East and North Africa and the rest of Africa reported very high adherence to all preventive measures in this study, except for downloading the contact tracing app. It is possible that these groups overreported adherence to preventive measures due to social desirability. However, persons originating from the Middle East and Africa also had the highest incidence of Covid-19 in Finland[29], therefore it is possible that these groups were more motivated to follow recommendations due to higher perceived risk of Covid-19 infection.

For peer review only - http://bmjopen.bmj.com/site/about/guidelines.xhtml

Women reported adherence to preventive measures more often than men both in the general and migrant origin populations, which is in consistency with previous studies[23]. Also in other areas of health and health-related behaviours, women generally have healthier habits than men[17, 30]. Findings of a positive association of older age with improved hand and coughing hygiene, use of face mask, and keeping a safety distance to others in the general population is also in consistency with previous studies[23, 31]. In contrast, among the migrant origin population, older age was associated with being less likely to stay at home with flu symptoms. The reasons for this should be explored in future studies. Migrant origin populations reported downloading the contact tracing app less frequently than the general population, and the inverse association of older age with downloading the app are in consistency with findings from UK[32-33]. Some of the participants indicated distrust towards authorities as a reason for not downloading the app, which is a concern raised among migrant origin populations also in previous studies[34]. Higher education was associated with higher likelihood of downloading the contact tracing app both among the general and migrant origin populations. The observed differences in how sociodemographic characteristics are associated with adherence to preventive measures in migrant origin compared with the general population point to a need for tailoring population-level measures for them to be effective in influencing behaviours of diverse population groups.

In the current study, little association between perceived access to information and adherence to preventive measures was observed. Both perceived access to information and self-reported adherence to preventive measures were high, which may explain lack of the observed associations. However, when examined by region of origin, the association of adequate access to information and adherence to preventive measures was particularly observed among persons born in Africa (excl. North Africa). A large proportion of persons belonging to this group were of Somali origin, which is a very close-knit community. Over the course of Covid-19, Somali origin community representatives and non-governmental associations were very active in distributing information within the community. Also the Somali-speaking TV channel was active in disseminating information on Covid-19. Other possible reasons for the observed association should be explored in further studies. During the Covid-19 pandemic in Finland, social listening was introduced to gather signals from diverse population groups on their perceptions and needs related to the crisis[35]. Multilingual crisis communications were developed in close collaboration with regional health and welfare professionals, key community representatives and migrant origin populations. In addition to these, Covid-19 incidence was regularly followed and the MigCOVID Survey, which constitutes the basis for this study was launched to gather population-based data on experiences during Covid-19 among migrant origin populations. When further developing future crisis preparedness, it may be worthwhile to consider use of additional systematic theory-driven tools, such as the framework for evaluating the effectiveness and sustainability of behaviourally and culturally informed interventions in complex settings, recently launched by the World Health Organisation[36].

Significant strengths of the current study are its population-based study design, representative sample, and availability of the general population reference group. The survey was available in 18 of the most commonly spoken languages in Finland, reducing language barriers for majority of the participants. Participation barriers were further reduced through provision of the questionnaire in both electronic and paper-based format, as well as by providing an alternative to respond via a telephone interview. Some limitations also need to be addressed. Self-reported access to information does not provide information on the quality of the used sources or whether the information was understood and applied as intended. There may be some report bias in the self-reported adherence to preventive measures due to social desirability. It may also be that respondents reported adherence to preventive measures, even though they may have not

For peer review only - http://bmjopen.bmj.com/site/about/guidelines.xhtml

adhered to them in all situations. While the sample size and participation rate were substantial compared with other surveys conducted among migrant origin population during Covid-19, not all of the analyses could have been performed by region of origin.

In conclusion, this study provided evidence on importance of multilingual communications in securing equity in access to information among migrant origin populations in crisis situations. While policy and practice acknowledging the increasing cultural and linguistic diversity of populations has been evidently lacking prior to the pandemic, a number of countries have developed measures to improve access to up-to-date crisis communications in multiple languages during Covid-19. These measures need to be carefully documented and synthesised to identify best practices and to encourage mutual learning, as well as to facilitate better preparedness in future crises. Population diversity is a global trend that is accelerating rapidly. Therefore, it is imperative that multilingual and crisis communications are included in crisis preparedness plans. Countries must be ready to produce crisis communications in multiple languages without a significant time lag from the onset of a crisis to ensure adequate access to information among diverse population groups. Findings on differences in sociodemographic characteristics associated with access to information and adherence to preventive measures across migrant origin populations and also in comparison with the general population highlight the need for acknowledging not only population's linguistic, but also cultural diversity. It is imperative to apply comprehensive theory-driven monitoring and impact assessment measures to analyse the needs, perceptions, and attitudes of diverse population groups during crisis situations to gain insights on drivers of behaviours, as well as to identify most effective strategies for influencing behaviors.

For peer review only

Author contributions: NS conceptualised the paper. NS drafted the manuscript with assistance of TP. TP conducted the literature review. EL conducted the statistical analyses. PK and AC have provided their critical comments for developing the manuscript. All authors have participated in interpreting the results and reviewing drafts. All authors have read and approved the manuscript sent for publication.

Funding: The MigCOVID Survey and the FinHealth 2017 follow-up Study were funded from the Finnish parliament's additional budget allocated for COVID-19 research. NS and EL received funding from the Coping of disabled persons and persons of migrant origin in exceptional and crisis situations – Building the future based on experiences during Covid-19 (Building the Future) project supported by the European Social Fund (grant number S22389). TP was funded by the Impact of coronavirus epidemic on wellbeing among foreign born population (MigCOVID) project. The funding bodies had no role in study design; in the collection, analysis and interpretation of data; in the writing of the report; or in the decision to submit the article for publication.

Acknowledgements: The authors would like to acknowledge all of the participants in the MigCOVID Survey and the Health 2017 Study, fieldwork personnel, as well as the experts involved in planning and data management of the studies.

Data sharing statement: The MigCOVID Survey data can be shared with other researchers within EU and ETA countries following an accepted research plan. More information: thl.fi/migcovid.

Conflict of interest: None

Ethics approval: All participants were made aware of the voluntary nature of participation in the surveys and that by participating, they permitted the use of their personal information according to the data protection notification on handling personal data. The MigCOVID Survey received ethical permission from the Ethical Committee of THL (THL/4061/6.02.01/2020). The FinHealth 2017 follow-up Study received ethical permission from the Ethics Committee II of the Helsinki and Uusimaa hospital region (HUS/2391/2020).

For peer review only

1 Diaz E, Norredam M, Aradhya S, et al. Situational brief: Migration and COVID-19 in Scandinavian countries, *Econ Geogr* 2021.

2 Li SL, Pereira RHM, Prete CA, et al. Higher risk of death from COVID-19 in low-income and non-White populations of Sao Paulo, Brazil, *BMJ Glob Health* 2021;6:10.1136/bmjgh-004959 doi:e004959 [pii] [published Online First: April 01].

3 Hayward SE, Deal A, Cheng C, et al. Clinical outcomes and risk factors for COVID-19 among migrant populations in high-income countries: A systematic review, *J Migr Health* 2021;3:100041 doi:10.1016/j.jmh.2021.100041 [doi].

4 Skogberg N, Koponen P, Lilja E, et al. Impact of Covid-19 on the health and wellbeing of persons who migrated to Finland: The MigCOVID Survey 2020-2021, 2021.

5 Schmidt H, Wild E, Schreyögg J. Explaining variation in health information seeking behaviour – Insights from a multilingual survey, *Health policy (Amsterdam)* 2021;125:618-26 doi:10.1016/j.healthpol.2021.01.008 [published Online First: May].

6 Massaro M, Tamburro P, La Torre M, et al. Non-pharmaceutical Interventions and the Infodemic on Twitter: Lessons Learned from Italy during the Covid-19 Pandemic, *J Med Syst* 2021;45:50-7 doi:10.1007/s10916-021-01726-7 [doi] [published Online First: March 06].

7 Brønholt RLL, Langer Primdahl N, Jensen AMB, et al. "I Just Want Some Clear Answers": Challenges and Tactics Adopted by Migrants in Denmark When Accessing Health Risk Information about COVID-19, *Int J Environ Res Public Health* 2021;18:8932 doi:10.3390/ijerph18178932 [published Online First: Aug 25,].

8 Finell E, Tiilikainen M, Jasinskaja-Lahti I, et al. Lived Experience Related to the COVID-19 Pandemic among Arabic-, Russian- and Somali-Speaking Migrants in Finland, *Int J Environ Res Public Health* 2021;18:10.3390/ijerph18052601 doi:2601 [pii] [published Online First: March 05].

9 Knights F, Carter J, Deal A, et al. Impact of COVID-19 on migrants' access to primary care and implications for vaccine roll-out: a national qualitative study, *Br J Gen Pract* 2021;71:e583-95 doi:10.3399/BJGP.2021.0028 [doi] [published Online First: July 29].

10 Deal A, Hayward SE, Huda M, et al. Strategies and action points to ensure equitable uptake of COVID-19 vaccinations: A national qualitative interview study to explore the views of undocumented migrants, asylum seekers, and refugees, *J Migr Health* 2021;4:100050 doi:10.1016/j.jmh.2021.100050 [doi].

11 Wang C, Tian Q, Zhao P, et al. Disease knowledge and attitudes during the COVID-19 epidemic among international migrants in China: a national cross-sectional study, *Int J Biol Sci* 2020;16:2895-905 doi:10.7150/ijbs.47075 [doi] [published Online First: September 04].

12 Valeriani G, Vukovic IS, Mollica R. Unconventional Answers to Unprecedented Challenges: The Swedish Experience During the COVID-19 Outbreak, *J Prev Med Public Health* 2020;53:233-5.

13 Nezafat Maldonado BM, Collins J, Blundell HJ, et al. Engaging the vulnerable: A rapid review of public health communication aimed at migrants during the COVID-19 pandemic in Europe, *J Migr Health* 2020;1:100004 doi:10.1016/j.jmh.2020.100004 [doi].

14 Skogberg N, Hussein I, Castaneda A. Impact of Covid-19 human mobility restrictions on migrant origin population in Finland, *in book: COVID-19 and Migration: Understanding Pandemic and Human Mobility.Published* 2020;11.

15 Skogberg N, Koponen P, Lilja E, et al. Access to information, preventive measures and working conditions during the coronavirus epidemic: Findings of the population-based MigCOVID Survey among persons who have migrated to Finland, 2021.

For peer review only - http://bmjopen.bmj.com/site/about/guidelines.xhtml

16 Madar AA, Benavente P, Czapka E, et al. COVID-19: information access, trust and adherence to health advice among migrants in Norway, *Archives of public health = Archives belges de santé publique* 2022;80:15 doi:10.1186/s13690-021-00764-4 [published Online First: Jan 4,].

17 Kuusio H, Seppänen A, Jokela S, et al. Ulkomaalaistaustaisten terveys ja hyvinvointi Suomessa: FinMonik-tutkimus 2018-2019, 2020.

18 Borodulin K, Sääksjärvi K. FinHealth 2017 Study: Methods, 2019. Finnish Institute for health and Welfare, THL; Helsinki.

19 United Nations. Standard country or area codes for statistical use (M49). 2021.

20 Graubard BI, Korn EL. Predictive margins with survey data, *Biometrics* 1999;55:652-9.

21 Lehtonen R, Pahkinen E. Practical methods for design and analysis of complex surveys: John Wiley & Sons 2004.

22 Robins JM, Rotnitzky A, Zhao LP. Estimation of regression coefficients when some regressors are not always observed, *J Am Stat Assoc* 1994;89:846-66.

23 Varghese NE, Sabat I, Neumann-Böhme S, et al. Risk communication during COVID-19: A descriptive study on familiarity with, adherence to and trust in the WHO preventive measures, *PloS one* 2021;16:e0250872.

24 Demirci Ş, Uğurluoğlu Ö, Konca M, et al. Socio-demographic characteristics affect health information seeking on the Internet in Turkey, *Health Info Libr J* 2021;38:304-12.

25 Nivette A, Ribeaud D, Murray A, et al. Non-compliance with COVID-19-related public health measures among young adults in Switzerland: Insights from a longitudinal cohort study, *Social science & medicine (1982)* 2021;268:113370 doi:10.1016/j.socscimed.2020.113370 [published Online First: Jan].

26 Gosselin A, Melchior M, Carillon S, et al. Deterioration of mental health and insufficient Covid-19 information among disadvantaged immigrants in the greater Paris area, *J Psychosom Res* 2021;146:110504 doi:S0022-3999(21)00149-5 [pii] [published Online First: July 01].

27 Margraf J, Brailovskaia J, Schneider S. Adherence to behavioral Covid-19 mitigation measures strongly predicts mortality, *PloS one* 2021;16:e0249392 doi:10.1371/journal.pone.0249392.

28 Margraf J, Brailovskaia J, Schneider S. Behavioral measures to fight COVID-19: An 8-country study of perceived usefulness, adherence and their predictors, *Plos one* 2020;15:e0243523.

29 Holmberg V, Salmi H, Kattainen S, et al. Association between first language and SARS-CoV-2 infection rates, hospitalization, intensive care admissions and death in Finland: a population-based observational cohort study, *Clin Microbiol Infect* 2022;28:107-13 doi:10.1016/j.cmi.2021.08.022 [published Online First: Jan].

30 Koponen P, Borodulin K, Lundqvist A, et al. Terveys, toimintakyky ja hyvinvointi Suomessa: FinTerveys 2017 -tutkimus. Terveyden ja hyvinvoinnin laitos, raportti 4/2018.

31 Bearth A, Luchsinger L, Siegrist M. Reactions of older Swiss adults to the COVID-19 pandemic: A longitudinal survey on the acceptance of and adherence to public health measures, *Soc Sci Med* 2021;280:114039.

32 Dowthwaite L, Fischer J, Vallejos EP, et al. Public Adoption of and Trust in the NHS COVID-19 Contact Tracing App in the United Kingdom: Quantitative Online Survey Study, *J Med Internet Res* 2021;23:e29085.

33 Skarpa PE, Garoufallou E. Information seeking behavior and COVID-19 pandemic: A snapshot of young, middle aged and senior individuals in Greece, *Int J Med Inf* 2021;150:104465.

For peer review only - http://bmjopen.bmj.com/site/about/guidelines.xhtml

34 Abba-Aji M, Stuckler D, Galea S, et al. Ethnic/racial minorities' and migrants' access to COVID-19 vaccines: A systematic review of barriers and facilitators. *J Migr Health* 2022:100086.

35 Lohiniva A, Sibenberg K, Austero S, et al. Social Listening to Enhance Access to Appropriate Pandemic Information Among Culturally Diverse Populations: Case Study From Finland *JMIR Infodemiology* 2022;2(2):e38343 doi: 10.2196/38343

36 World Health Organization. Guide to evaluating behaviourally and culturally informed health interventions in complex settings. 2022. https://www.who.int/europe/publications/i/item/WHO-EURO-2022-6045-45810-65956

For peer review only

For peer review only - http://bmjopen.bmj.com/site/about/guidelines.xhtml

STROBE 2007 (v4) Statement—Checklist of items that should be included in reports of *cross-sectional studies*

Section/Topic	Item #	Recommendation	Reported on page #
**Title and abstract**	1	(*a*) Indicate the study's design with a commonly used term in the title or the abstract	1,2
		(*b*) Provide in the abstract an informative and balanced summary of what was done and what was found	2
**Introduction**			
Background/rationale	2	Explain the scientific background and rationale for the investigation being reported	4
Objectives	3	State specific objectives, including any prespecified hypotheses	4
**Methods**			
Study design	4	Present key elements of study design early in the paper	5
Setting	5	Describe the setting, locations, and relevant dates, including periods of recruitment, exposure, follow-up, and data collection	5
Participants	6	(*a*) Give the eligibility criteria, and the sources and methods of selection of participants	5
Variables	7	Clearly define all outcomes, exposures, predictors, potential confounders, and effect modifiers. Give diagnostic criteria, if applicable	5,6
Data sources/ measurement	8*	For each variable of interest, give sources of data and details of methods of assessment (measurement). Describe comparability of assessment methods if there is more than one group	5,6
Bias	9	Describe any efforts to address potential sources of bias	*6*
Study size	10	Explain how the study size was arrived at	5
Quantitative variables	11	Explain how quantitative variables were handled in the analyses. If applicable, describe which groupings were chosen and why	5,6
Statistical methods	12	(*a*) Describe all statistical methods, including those used to control for confounding	6
		(*b*) Describe any methods used to examine subgroups and interactions	6
		(*c*) Explain how missing data were addressed	6
		(*d*) If applicable, describe analytical methods taking account of sampling strategy	6
		(*e*) Describe any sensitivity analyses	-
**Results**			

For peer review only

Participants	13*	(a) Report numbers of individuals at each stage of study—eg numbers potentially eligible, examined for eligibility, confirmed eligible, included in the study, completing follow-up, and analysed	5
		(b) Give reasons for non-participation at each stage	5
		(c) Consider use of a flow diagram	-
Descriptive data	14*	(a) Give characteristics of study participants (eg demographic, clinical, social) and information on exposures and potential confounders	16
		(b) Indicate number of participants with missing data for each variable of interest	16-19
Outcome data	15*	Report numbers of outcome events or summary measures	16-19
Main results	16	(*a*) Give unadjusted estimates and, if applicable, confounder-adjusted estimates and their precision (eg, 95% confidence interval). Make clear which confounders were adjusted for and why they were included	16-19
		(*b*) Report category boundaries when continuous variables were categorized	-
		(*c*) If relevant, consider translating estimates of relative risk into absolute risk for a meaningful time period	-
Other analyses	17	Report other analyses done—eg analyses of subgroups and interactions, and sensitivity analyses	8
**Discussion**			
Key results	18	Summarise key results with reference to study objectives	8
Limitations	19	Discuss limitations of the study, taking into account sources of potential bias or imprecision. Discuss both direction and magnitude of any potential bias	9
Interpretation	20	Give a cautious overall interpretation of results considering objectives, limitations, multiplicity of analyses, results from similar studies, and other relevant evidence	8,9
Generalisability	21	Discuss the generalisability (external validity) of the study results	10
**Other information**			
Funding	22	Give the source of funding and the role of the funders for the present study and, if applicable, for the original study on which the present article is based	11

*Give information separately for cases and controls in case-control studies and, if applicable, for exposed and unexposed groups in cohort and cross-sectional studies.

Note: An Explanation and Elaboration article discusses each checklist item and gives methodological background and published examples of transparent reporting. The STROBE checklist is best used in conjunction with this article (freely available on the Web sites of PLoS Medicine at http://www.plosmedicine.org/, Annals of Internal Medicine at http://www.annals.org/, and Epidemiology at http://www.epidem.com/). Information on the STROBE Initiative is available at www.strobe-statement.org.

For peer review only

BMJ Open

The association of sociodemographic characteristics with access to Covid-19 information and adherence to preventive measures among migrant origin and general populations in Finland: a cross-sectional study

Journal:	*BMJ Open*
Manuscript ID	bmjopen-2022-069192.R1
Article Type:	Original research
Date Submitted by the Author:	02-Feb-2023
Complete List of Authors:	Skogberg, N. ; National Institute for Health and Welfare, Helsinki, Finland, Welfare<br>Prinkey, Tyler; Finnish Institute for Health and Welfare<br>Lilja, Eero; National Institute for Health and Welfare, Welfare<br>Koponen, Päivikki; National Institute for Health and Welfare, Public Health Solutions<br>Castaneda, Anu; Terveyden ja hyvinvoinnin laitos,
**Primary Subject Heading**:	Public health
Secondary Subject Heading:	Communication, Epidemiology, Evidence based practice, Global health, Health policy
Keywords:	COVID-19, Public health < INFECTIOUS DISEASES, PUBLIC HEALTH

SCHOLARONE™
Manuscripts

I, the Submitting Author has the right to grant and does grant on behalf of all authors of the Work (as defined in the below author licence), an exclusive licence and/or a non-exclusive licence for contributions from authors who are: i) UK Crown employees; ii) where BMJ has agreed a CC-BY licence shall apply, and/or iii) in accordance with the terms applicable for US Federal Government officers or employees acting as part of their official duties; on a worldwide, perpetual, irrevocable, royalty-free basis to BMJ Publishing Group Ltd ("BMJ") its licensees and where the relevant Journal is co-owned by BMJ to the co-owners of the Journal, to publish the Work in this journal and any other BMJ products and to exploit all rights, as set out in our licence.

The Submitting Author accepts and understands that any supply made under these terms is made by BMJ to the Submitting Author unless you are acting as an employee on behalf of your employer or a postgraduate student of an affiliated institution which is paying any applicable article publishing charge ("APC") for Open Access articles. Where the Submitting Author wishes to make the Work available on an Open Access basis (and intends to pay the relevant APC), the terms of reuse of such Open Access shall be governed by a Creative Commons licence – details of these licences and which Creative Commons licence will apply to this Work are set out in our licence referred to above.

Other than as permitted in any relevant BMJ Author's Self Archiving Policies, I confirm this Work has not been accepted for publication elsewhere, is not being considered for publication elsewhere and does not duplicate material already published. I confirm all authors consent to publication of this Work and authorise the granting of this licence.

For peer review only

Title: The association of sociodemographic characteristics with access to Covid-19 information and adherence to preventive measures among migrant origin and general populations in Finland: a cross-sectional study

Natalia Skogberg*[a], Tyler Prinkey[a], Eero Lilja[a], Päivikki Koponen[a], Anu E Castaneda[a]. [The surname of each author is underlined]

*Corresponding author; natalia.skogberg@thl.fi; ORCHID: 0000-0002-8829-4159. Address: Finnish Institute for Health and Welfare, Mannerheimintie 166, P.O. box 27, 00271 Helsinki. Tel. +358 29 524 7916.

[a]Department of Public Health and Welfare, Finnish institute for Health and Welfare, Helsinki, Finland.

word count: 4346

tables: 5

For peer review only - http://bmjopen.bmj.com/site/about/guidelines.xhtml

Abstract

Objectives: This study examines how access to Covid-19 information and adherence to preventive measures varies by sociodemographic characteristics, and whether the associations differ among the migrant origin and the general Finnish population. Additionally, the association of perceived access to information with adherence to preventive measures is examined.

Design: Cross-sectional, population-based random sample.

Background: Equity in access to information is crucial for securing individual wellbeing and successful management of a crisis at population level.

Setting: Persons who have a residence permit in Finland.

Participants: Migrant origin population constitutes of persons born abroad aged 21 to 66 years, who took part in the MigCOVID Survey (n=3 668). Participants in the Health 2017 follow-up survey, representing the general Finnish population consitituted the reference group (n=3 490).

Outcome measures: Self-perceived access to Covid-19 information, adherence to preventive measures.

Results: Self-perceived access to information and adherence to preventive measures was overall high both among the migrant origin and the general population. Perceived adequate access to information was associated with living in Finland for 12 years or longer (OR 1.94, 95%CI 1.05-3.57) and excellent Finnish/Swedish language skills (OR 2.71, 95% CI 1.62-4.53) among the migrant origin population and with higher education (OR 3.56, 95% CI 1.49-8.55 for tertiary and OR 2.87, 95%CI 1.25-6.59 for secondary) among the general population. The association between examined sociodemographic characteristics with adherence to preventive measures varied by study group.

Conclusions: Findings on the association of perceived access to information with language proficiency in official languages highlight the need for rapid multilingual and simple language crisis communications. Findings also suggest that crisis communications and measures designed to influence health behaviours at population level may not be directly transferable if the aim is to influence health behaviours also among ethnically and culturally diverse populations.

key words: migrant, Covid-19, communication, MigCOVID, prevention

For peer review only - http://bmjopen.bmj.com/site/about/guidelines.xhtml

Strengths and limitations of the study

- Significant strengths of the current study are its population-based study design, representative sample, and availability of the general population reference group.
- Barriers to participation were reduced by making the survey material available in 18 languages both in electronic and paper-based format, as well as by providing with the option to respond via a telephone interview conducted by multilingual interviewers.
- One limitation is that self-reported access to information does not provide information on the quality of the used sources or whether the information was understood and applied as intended.
- There may be some report bias in the self-reported adherence to preventive measures due to social desirability. It may also be that respondents reported adherence to preventive measures, even though they may have not adhered to them in all situations.

For peer review only

For peer review only - http://bmjopen.bmj.com/site/about/guidelines.xhtml

BACKGROUND

Covid-19 exposed the weakest spots in crisis preparedness, highlighting the evident lack of policies and practices that acknowledge increasing globalization and population cultural diversity[1]. Vulnerabilities of migrant origin populations were reflected in significantly higher incidence of Covid-19 infections, higher risk for hospitalizations, mortality rates, as well as higher economic, social, and psychological impact of the pandemic compared with general populations[2-4]. Information guidance was crucial in management of the pandemic, however crisis communications were challenged by ever-evolving information and changes in official guidelines[5- 6]. From the onset of the pandemic, significant concerns were raised regarding equity in access to information among migrant origin populations. Difficulties in accessing up-to-date and reliable information occurred due to language barriers, lack of trust in the health system, and gaps in health and digital literacy[7-9]. Inequities in access to information may lead to stress, lower awareness, and hence also lower adherence to preventive measures[7,10-11]. In addition to impact on individual-level coping in a crisis, these may negatively impact overall crisis management and hence may jeopardise societal security.

While multilingual communications and community outreach programmes were launched in a number of countries to improve equity in access to information across different population groups[12], practices across countries varied. During the first half year of the pandemic, only half of European countries had information on Covid-19 available in at least one foreign language, and only several countries had information in multiple foreign languages[13]. Significant efforts have been placed to develop multilingual Covid-19 communications also in Finland[14]. Finland is a country in North of Europe, with the population of 5.6 million. Migration to Finland rapidly accelerated over the past decades, from 0.5% of the total population in the 1990 to 8% in 2021[15]. In 2021, the largest migrant origin populations in Finland were persons born in the former Soviet Union, Estonia, Russian Federation, Sweden, Irak, China, and Somalia. A few weeks into the pandemic, the Finnish Institute for Health and Welfare (THL) introduced information on Covid-19 in 16 foreign languages. Language selection was later expanded by several additional languages. In addition to THL that produced crisis communications at the national level, regional health authorities and non-governmental organisations also produced multilingual communications. It was well acknowledged that simply publishing multilingual information is not sufficient, and particular efforts were taken to ensure effective information dissemination to the target group. Dissemination of national-level Covid-19 communications was implemented by THL's Multilingual and multichannel communications task force in close collaboration with regional health and social welfare professionals, non-governmental organisations, religious community leaders, and other key community representatives[16].

Because multilingual Covid-19 comminications were available with a slight delay, migrant origin populations in Finland initially perceived they did not receive sufficient information, but the situation improved over the course of the pandemic[8]. To date, studies exploring perceived access to information and adherence to preventive measures among migrant origin populations have been generally based on qualitative and non-representative samples[7-9, 17]. The general population reference group is rarely available, which makes contextualisation of the findings challenging. There is also limited information on how access to information and adherence to preventive measures has varied by sociodemographic characteristics, and whether these differed between migrant origin and the general population. Furthermore, there is limited information on how perceived access to information has impacted behaviours in different population groups. This information is needed for evaluation of crisis response measures and for identifying groups at highest risk of vulnerabilities in crisis situations. These all significantly contribute to development of preparedness in future crises. The aim of the current study is to fill this gap in knowledge by using

For peer review only - http://bmjopen.bmj.com/site/about/guidelines.xhtml

representative population-based data to examine how access to Covid-19 information and adherence to preventive measures varied by sociodemographic characteristics, and whether these differed between the migrant origin and the general Finnish populations. Additionally, the association of access to information with adherence to preventive measures is examined.

METHODS

The present study is based on the Impact of the Coronavirus on the Wellbeing of the Foreign Born Population (MigCOVID) Survey. The survey was conducted between October 2020 and February 2021, coinciding with the second wave of the Covid-19 pandemic in Finland. A more detailed description of the survey can be found elsewhere[4]. Briefly, the MigCOVID Survey was a 2-year follow-up study to the Survey on Wellbeing among Foreign Born Population (FinMonik) [18]. The original stratified random sample was drawn from the Finnish Population Register using the following selection criteria: age 18-64 years, both the person belonging to the sample and their parents were born outside of Finland, and residence in Finland for at least one year. Following exclusion of persons declining further contact during the FinMonik Survey and who no longer lived in Finland, the updated sample of the MigCOVID Survey comprised of 5 269 persons aged 20 to 66 years. In addition to including participants in the FinMonik Survey, a supplementary sample of 982 persons of Somali origin was drawn using simple random sampling and following the same selection criteria as the original sample. Following exclusion of overcoverage (n=112), the final sample of the MigCOVID Survey constituted of 6 139 persons. Altogether 3 368 persons took part in the MigCOVID Survey, with a participation rate of 60%.

Invitation letters were sent by mail with personal login information to the electronic questionnaire. Data collection was supplemented with mailed paper questionnaires and telephone interviews. Invitation letters, information about the survey, and questionnaires were available in 18 languages. Telephone interviews were carried out by trained multilingual fieldwork personnel in 12 languages. A reference group was formed from a sub-population of the FinHealth 2017 follow-up Study of corresponding age to the participants in the MigCOVID Survey. As the youngest participants in the FinHealth 2017 follow-up Study were 21 years, the reference group of this study constitutes of persons aged 21 to 66 years (n=3 490 participated, participation rate 51%). The FinHealth 2017 Study had a nationally representative, two-stage stratified cluster sample of adults living in Finland[19]. The study was conducted within the same time frame and following a similar protocol to the MigCOVID Survey.

Patient and public involvement in research

This study does not involve patients. The public were not involved in the planning or design of the research. Data collection was implemented by trained multilingual fieldwork personnel. Findings have been disseminated to the public in multiple languages through the webpages of the survey and social media. Additionally, group discussions with representatives of different migrant origin populations have been conducted to disseminate and to hear feedback on the findings.

Variable definitions

Information on date of birth, age, sex, and length of residence in Finland was obtained from the Finnish Population Register. The MigCOVID Survey participants were grouped into six regions of origin based on their country of birth following a slightly modified United Nations area code grouping[20]: Russia or the former Soviet Union (FSU); Estonia; the rest of Europe, North America, and Oceania; the Middle East and North Africa; the rest of Africa (later in the text as Africa); Asia or other (later in the text as Asia). In the

For peer review only - http://bmjopen.bmj.com/site/about/guidelines.xhtml

latter group, "other" constituted of persons born in Latin America (n=96). Information on highest completed level of education in Finland or abroad, economic activity, and Finnish/Swedish language skills (official languages of Finland) was gathered as self-report measures during the survey. Categorization of the included sociodemographic characteristics can be found in Table 1.

Self-perceived access to information was assessed in both studies with the question: Have your received adequate information on how to avoid getting infected with the coronavirus and how to prevent it from spreading? The answer options were: I have not received any information or the information I have received has been completely inadequate; I have received information, but I would have needed more; or I have received adequate information. The first two answer options were grouped.

Adherence to preventive measures was based on the measures recommended by THL at the point of finalization of the questionnaire in September 2020. Participants were asked: Which measures have you taken to avoid getting infected with the coronavirus and prevent it from spreading: I wash my hands more frequently; I use sanitisers more frequently; I take care of hygiene when coughing (e.g. coughing into a disposable tissue, not coughing into hands; I stay at home if I have flu symptoms (e.g. cough, cold symptoms or sore throat); I wear a single use mask or cloth mask during free time (when it is not possible to avoid contact with other people); I keep a safe distance to other people outside of the home; I do not shake hands with the people I meet. The answer options were: Yes, I follow the instruction/ recommendation, and I do not follow the instruction/recommendation. The questions on washing hands, use of sanitisers and coughing hygiene were grouped into a joint variable "improved hand and coughing hygiene". Answers were dichotomized as "yes" (yes to all three) and "no" (no to at least one measure).

Participants were also asked if they have downloaded the Koronavilkku contact tracing app. In the MigCOVID Survey, the answer options were: yes; no, because the app is not available for my phone; no, because I don't know what it is; no, because the app is not available in the languages that I speak; no, for other reasons, what? The answers were dichotomised as yes/no. The open-ended answers were analysed using thematic grouping. In the Health 2017 follow-up Study, the answer options were: yes; no, because I don't have a suitable smartphone; no for other reasons. These were also dichotomized as yes or no.

Statistical analyses

The differences between groups were tested using logistic regression. P-value based on Satterthwaite-adjusted F-value, as well as odds ratio and its 95% confidence interval were used to assess the differences. The model-adjusted proportions were calculated with predictive margins[21]. The stratification of the sample was accounted for in all analyses and finite population correction was applied[22]. The data were weighted using inverse probability weights to reduce the non-response bias and to take varying sampling probabilities into account[23]. The calculation of the IPWs is described elsewhere[4, 19]. All analyses were carried out with SAS 9.4 and SUDAAN 11.0.3 statistical software.

For peer review only

RESULTS

Descriptive characteristics of the study participants are presented in Table 1. The proportion of men was lowest among persons born in Russia/FSU and highest among persons born in the Middle East and North Africa. Persons born in Africa ~~(excl. North Africa)~~ were the youngest. The proportion of those with at least tertiary education was lowest among persons born in Estonia and highest among persons born in the rest of Europe, North America and Oceania. Employment was lowest among persons born in the Middle East and North Africa. Persons born in Russia/FSU had lived in Finland the longest and had the highest Finnish/Swedish language proficiency.

[Table 1 about here]

Perceived access to information and adherence to preventive measures among the study participants is presented in Table 2. Migrant origin population reported receiving fully adequate Covid-19 information less frequently than the general population. Perceived access to information was lowest among persons born in Europe (excl. Russia/FSU, Estonia), North America and Oceania, the Middle East and North Africa, and Asia.

[Table 2 about here]

Persons born in Russia/FSU and Estonia reported lower, while persons born in the Middle East and Africa reported higher adherence to preventive measures compared with the general population. Migrant origin population reported downloading the Koronavilkku contact tracing app less frequently than the general population. The most common reasons mentioned for not downloading the app were not knowing about it; the app was unnecessary; not wanting to; the app would just increase stress; mistrust towards the authorities; technical difficulties; and that the app consumes too much phone memory. (results not shown)

The association of sociodemographic characteristics with self-perceived access to information and adherence to Covid-19 preventive measures among the general population is presented in Table 3. Higher education was positively associated with self-perceived sufficient access to information. Women were more likely than men to report adhering to all other preventive measures except for keeping a safety distance to others. Older age was positively associated with improved hand hygiene and use of a face mask. Additionally, persons in the oldest age group (50-66 years) were more likely to keep a safety distance to others but less likely to download the contact tracing app compared with the youngest (21-34 years) age group. Persons with tertiary education were more likely to report the use of a face mask than persons with basic education. Higher education and employment were also associated with downloading the contact tracing app.

[Table 3 about here]

For peer review only - http://bmjopen.bmj.com/site/about/guidelines.xhtml

Table 1 Descriptive characteristics of the study participants

	General population	Born abroad total	Russia or former Soviet Union	Estonia	Rest of Europe, North America, Oceania	Middle East and North Africa	Rest of Africa	Asia
	n=6543	n=3456	n=1115	n=300	n=712	n=436	n=302	n=746
	N (%)	N (%)	N (%)	N (%)	N (%)	N (%)	N (%)	N (%)
**Men**	1604 (50.5)	1614 (51.6)	380 (39.0)	110 (46.5)	399 (64.7)	286 (68.8)	174 (54.9)	265 (42.7)
**Age**								
21–34 years	533 (29.3)	1090 (34.7)	251 (24.5)	56 (25.0)	179 (32.5)	178 (49.7)	156 (45.7)	270 (38.1)
35–49 years	1142 (33.6)	1509 (41.7)	412 (38.0)	119 (39.7)	339 (48.1)	178 (36.2)	122 (46.9)	339 (43.0)
50–66 years	1815 (37)	1012 (23.6)	452 (37.5)	125 (35.3)	194 (19.4)	80 (14.1)	24 (7.4)	137 (18.9)
**Education**								
Basic education or less	273 (6.5)	380 (13.2)	57 (6.0)	32 (16.0)	55 (11.8)	86 (16.8)	56 (21.0)	94 (14.2)
Secondary education	1764 (49.7)	1276 (46.1)	435 (56.6)	147 (59.2)	210 (33.5)	160 (50.8)	117 (42.4)	207 (36.2)
Tertiary education or higher	1412 (43.8)	1888 (40.8)	609 (37.4)	113 (24.8)	440 (54.6)	174 (32.4)	119 (36.6)	433 (49.6)
**Economic activity**								
Employed full-time/part-time	2404 (70.7)	2299 (65.7)	699 (64.8)	218 (70.9)	504 (72.5)	197 (51.6)	172 (59.9)	509 (69.7)
Unemployed, student or other	1034 (29.3)	1229 (34.3)	396 (35.2)	77 (29.1)	195 (27.5)	225 (48.4)	119 (40.1)	217 (30.3)
**Length of residence in Finland**								
3–6.99 years	N/A	793 (17.9)	163 (6.7)	32 (6.5)	134 (22.6)	183 (31.8)	46 (14.3)	235 (24.0)
7–11.99 years	N/A	1020 (28.7)	273 (18.9)	124 (43.4)	200 (28.2)	116 (30.4)	58 (21.3)	249 (31.6)
12+ years	N/A	1798 (53.4)	679 (74.3)	144 (50.1)	378 (49.2)	137 (37.9)	198 (64.3)	262 (44.3)
**Excellent Finnish/Swedish language proficiency**	N/A	1364 (35.4)	480 (43.2)	182 (52.1)	273 (32.0)	122 (29.1)	163 (50.3)	144 (17.8)

For peer review only

Table 2 Perceived access to information and self-reported adherence to preventive measures among the study population[1]

	General population n=6543 % (95%CI)	Born abroad total n=3456 % (95%CI)	Russia or former Soviet Union n=1115 % (95%CI)	Estonia n=300 % (95%CI)	Rest of Europe, North America, Oceania n=712 % (95%CI)	Middle East and North Africa n=436 % (95%CI)	Rest of Africa n=302 % (95%CI)	Asia n=746 % (95%CI)
**Access to information**								
Received adequate information	97.1 (95.7–98.0)	**93.5 (92.0–94.8)*****	96.1 (94.3–97.4)	97.4 (94.0–98.9)	**91.4 (87.0–94.5)*****	**91.8 (86.6–95.1)****	93.9 (88.8–96.7)	**91.4 (86.8–94.5)*****
Received, but would have needed more	2.5 (1.6–3.9)	**5.3 (4.2–6.7)*****	2.8 (1.8–4.3)	1.9 (0.7–5.5)	**6.2 (3.8–9.9)****	**7.8 (4.6–13.0)****	5.4 (2.7–10.6)	**7.5 (4.8–11.6)*****
No information/completely inadequate	0.4 (0.2–0.8)	**1.1 (0.6–2.0)*****	**1.1 (0.5–2.4)***	0.6 (0.2–2.4)	**2.4 (0.9–6.2)****	0.4 (0.1–1.1)	0.7 (0.3–1.9)	1.1 (0.2–5.6)
**Following preventive measures**								
Staying home with flu symptoms	96.1 (94.6–97.2)	96.4 (95.1–97.4)	94.2 (91.4–96.1)	92.1 (85.2–95.9)	96.4 (92.8–98.2)	**99.2 (98.1–99.7)****	**99.0 (97.6–99.6)****	98.2 (95.2–99.3)
Improved hand and coughing hygiene	92.2 (90.3–93.7)	92.8 (91.0–94.2)	**85.3 (81.2–88.7)*****	**84.4 (75.7–90.4)***	**96.3 (92.7–98.1)***	95.7 (91.4–97.9)	**98.7 (96.9–99.5)*****	96.2 (92.4–98.1)
Use of face mask	81.9 (79.6–83.9)	81.5 (79.0–83.7)	**69.5 (64.1–74.4)*****	**65.0 (55.5–73.5)*****	84.2 (78.5–88.5)	**88.9 (83.5–92.7)***	**93.7 (90.4–96.0)*****	**89.2 (84.1–92.9)*****
Keeping a safety distance	94.6 (93.0–95.8)	93.5 (91.6–95.0)	92.0 (88.5–94.6)	**87.1 (78.1–92.8)****	92.6 (86.6–96.0)	**96.8 (93.0–98.6)**	**97.3 (95.3–98.5)***	94.9 (90.2–97.4)
Avoiding shaking hands with others	98.8 (98.3–99.2)	**93.2 (91.5–94.6)*****	**89.7 (86.2–92.3)*****	**84.7 (75.5–90.9)*****	**95.8 (92.5–97.7)*****	**95.4 (92.2–97.4)*****	**95.7 (92.5–97.6)*****	**96.2 (92.0–98.3)****
Downloaded Koronavilkku contact tracing app	62.4 (60.1–64.7)	**44.1 (41.2–47.2)*****	**33.5 (28.4–39.0)*****	**23.1 (16.6–31.2)*****	**53.5 (46.6–60.3)***	**51.4 (43.7–59.1)****	**50.8 (40.2–61.4)***	**52.8 (46.0–59.4)****

[1]Adjusted for age and sex

*p<0.05; **p<0.01; ***p<0.001, in comparison with the general population

For peer review only - http://bmjopen.bmj.com/site/about/guidelines.xhtml

Table 3 The association of sociodemographic characteristics with perceived adequate access to information and adherence to Covid-19 related preventive measures among the general population

	Perceived access to information	Staying home with flu symptoms	Improved hand and coughing hygiene	Use of face mask	Keeping a safety distance to others	Avoiding shaking hands with others	Downloaded Koronavilkku app
	OR (95%CI)	OR (95%CI)	OR (95%CI)	OR (95%CI)	OR (95%CI)	OR (95%CI)	OR (95%CI)
**Sex**[1]							
Men	1.00	1.00	1.00	1.00	1.00	1.00	1.00
Women	0.75 (0.36–1.55)	**2.53 (1.14–5.59)***	**2.43 (1.58–3.74)*****	**3.30 (2.49–4.38)*****	1.17 (0.68–2.02)	**2.46 (1.16–5.19)***	**1.64 (1.36–1.98)*****
**Age**[2]							
21–34 years	1.00	1.00	1.00	1.00	1.00	1.00	1.00
35–49 years	1.51 (0.59–3.89)	1.35 (0.61–3.00)	**1.92 (1.11–3.32)***	**1.50 (1.03–2.21)***	1.24 (0.65–2.39)	2.17 (0.90–5.25)	0.79 (0.58–1.07)
50–66 years	2.08 (0.80–5.36)	2.21 (0.99–4.91)	**2.08 (1.23–3.52)****	**2.05 (1.41–2.97)*****	**2.46 (1.27–4.79)****	1.96 (0.86–4.45)	**0.61 (0.45–0.81)*****
**Education**[3]							
Basic education or less	1.00	1.00	1.00	1.00	1.00	1.00	1.00
Secondary education	**2.87 (1.25–6.59)***	1.13 (0.48–2.69)	1.12 (0.37–3.36)	1.28 (0.88–1.87)	0.94 (0.43–2.04)	1.12 (0.37–3.36)	**2.13 (1.58–2.87)*****
Tertiary education or higher	**3.56 (1.49–8.55)****	1.51 (0.61–3.75)	1.19 (0.37–3.86)	**2.20 (1.41–3.44)*****	1.10 (0.49–2.47)	1.19 (0.37–3.86)	**4.48 (3.22–6.24)*****
**Economic activity**[3]							
Unemployed, student or other	1.00	1.00	1.00	1.00	1.00	1.00	1.00
Employed full-time/part-time	0.88 (0.37–2.09)	1.81 (0.86–3.83)	0.89 (0.40–1.96)	0.93 (0.66–1.30)	0.80 (0.40–1.59)	0.89 (0.40–1.96)	**0.63 (0.50–0.79)*****

[1]Adjusted for age
[2]Adjusted for sex
[3]Adjusted for age and sex
*p<0.05; **p<0.01; ***p<0.001

For peer review only - http://bmjopen.bmj.com/site/about/guidelines.xhtml

The association of sociodemographic characteristics with self-perceived access to information and adherence to Covid-19 preventive measures among the migrant origin population is presented in Table 4. Longer length of residence and excellent Finnish/Swedish skills were associated with perceived sufficient information on Covid-19. Women were generally more likely to adhere to preventive measures than men. Older age was associated with a lower likelihood of reporting staying at home with flu symptoms and downloading the Koronavilkku contact tracing app, while tertiary education was associated with a greater likelihood of downloading the Koronavilkku app. Secondary education was associated with being less likely, whereas excellent Finnish/Swedish skills were associated with being more likely to avoid shaking hands with others. Those who have lived in Finland for 7-12 years were less likely to use a face mask than those who have lived in Finland for a shorter period.

[Table 4 about here]

The association of self-perceived access to information with adherence to preventive measures is presented in Table 5. When the migrant origin population was examined as one group, the association was statistically significant for keeping a safety distance to others only. When examined by region of origin, statistically significant differences emerged in the population born in Africa (excl. North Africa) in case of all preventive measures except for downloading the Koronavilkku app. Because of widened conficence intervals in the subgroup analyses, interaction analyses were run to examine whether the associations for this group really differed from other regional groups. The p-values for the differences of Africa (excl. North Africa) group vs. other groups combined were p=0.101 for staying at home with flu symptoms; p=0.018 for following improved hand and coughing hygiene; p=0.074 for using a face mask; p=0.255 for keeping a safety distance to others; p=0.034 for avoiding shaking hands with others; and p=0.504 for downloading the Koronavilkku app. Based on these, it appears that the association of perceived access to information and following preventive measures was different for this regional group.

[Table 5 about here]

For peer review only - http://bmjopen.bmj.com/site/about/guidelines.xhtml

Table 4 The association of sociodemographic characteristics with perceived adequate access to information, adherence to Covid-19 related preventive measures, and Covid-19 infection among migrant origin persons

	Perceived access to information	Staying home with flu symptoms	Improved hand and coughing hygiene	Use of face mask	Keeping a safety distance to others	Avoiding shaking hands with others	Downloaded Koronavilkku app
	OR (95%CI)	OR (95%CI)	OR (95%CI)	OR (95%CI)	OR (95%CI)	OR (95%CI)	OR (95%CI)
**Sex[1]**							
Men	1.00	1.00	1.00	1.00	1.00	1.00	1.00
Women	1.22 (0.79–1.89)	1.07 (0.60–1.93)	**2.10 (1.33–3.31)****	**1.70 (1.23–2.33)****	**2.12 (1.26–3.56)****	**2.41 (1.48–3.92)*****	1.08 (0.84–1.39)
**Age[2]**							
21-34 years	1.00	1.00	1.00	1.00	1.00	1.00	1.00
35-49 years	0.94 (0.57–1.56)	**0.25 (0.14–0.47)*****	1.01 (0.58–1.76)	1.10 (0.76–1.59)	1.36 (0.73–2.52)	1.06 (0.62–1.82)	**0.61 (0.46–0.82)****
50-66 years	1.39 (0.73–2.66)	**0.24 (0.12–0.48)*****	0.93 (0.51–1.69)	1.30 (0.86–1.97)	2.09 (0.99–4.40)	1.29 (0.68–2.43)	**0.35 (0.25–0.49)*****
**Education[3]**							
Basic education or less	1.00	1.00	1.00	1.00	1.00	1.00	1.00
Secondary education	0.99 (0.50–1.94)	0.58 (0.14–2.40)	0.57 (0.22–1.46)	0.73 (0.42–1.25)	0.87 (0.36–2.10)	**0.38 (0.15–0.95)***	0.94 (0.63–1.41)
Tertiary education or higher	0.66 (0.34–1.27)	0.82 (0.19–3.56)	1.25 (0.49–3.20)	1.08 (0.62–1.88)	0.86 (0.36–2.07)	1.09 (0.42–2.86)	**1.72 (1.15–2.58)****
**Economic activity[3]**							
Unemployed, student or other	1.00	1.00	1.00	1.00	1.00	1.00	1.00
Employed full-time/part-time	0.69 (0.43–1.13)	1.18 (0.63–2.24)	1.21 (0.76–1.94)	1.11 (0.79–1.56)	1.58 (0.87–2.87)	0.87 (0.54–1.38)	0.92 (0.71–1.20)
**Length of residence in Finland[3]**							
3-6.99 years	1.00	1.00	1.00	1.00	1.00	1.00	1.00
7-11.99 years	1.15 (0.63–2.11)	0.93 (0.30–2.84)	0.50 (0.23–1.09)	**0.45 (0.27–0.75)****	1.21 (0.52–2.82)	0.68 (0.34–1.38)	0.92 (0.71–1.20)
12+ years	**1.94 (1.05–3.57)***	0.85 (0.31–2.30)	0.50 (0.25–1.00)	0.62 (0.38–1.01)	1.35 (0.64–2.84)	1.05 (0.56–1.97)	0.92 (0.71–1.20)
**Finnish/Swedish proficiency[3]**							
Intermediate/beginner or less	1.00	1.00	1.00	1.00	1.00	1.00	1.00
Excellent	**2.71 (1.62–4.53)*****	0.98 (0.51–1.86)	0.83 (0.52–1.30)	1.04 (0.76–1.44)	1.28 (0.72–2.26)	**1.73 (1.06–2.84)***	1.13 (0.87–1.47)

[1]Adjusted for age
[2]Adjusted for sex
[3]Adjusted for age and sex
*p<0.05; **p<0.01; ***p<0.001

For peer review only

Table 5 The association of adequate perceived access to information with following preventive measures by region of origin[1]

	Staying home with flu symptoms	Following improved hand and coughing hygiene	Using a face mask	Keeping a safety distance to others	Avoiding shaking hands with others	Downloading the Koronavilkku app
	OR (95%CI)	OR (95%CI)	OR (95%CI)	OR (95%CI)	OR (95%CI)	OR (95%CI)
General population	0.58 (0.08–4.31)	0.72 (0.23–2.24)	0.95 (0.46–1.97)	0.99 (0.27–3.59)	1.20 (0.16–9.00)	1.78 (0.90–3.52)
Born abroad total	1.58 (0.60–4.16)	0.73 (0.37–1.43)	1.49 (0.88–2.53)	**2.54 (1.15–5.61)***	1.53 (0.71–3.33)	1.03 (0.63–1.69)
Russia or former Soviet Union	1.01 (0.27–3.74)	1.25 (0.46–3.39)	2.87 (1.35–6.11)	2.51 (0.80–7.90)	1.57 (0.46–5.37)	2.05 (0.87–4.81)
Estonia	3.19 (0.44–23.28)	0.25 (0.04–1.77)	0.46 (0.08–2.62)	4.56 (0.56–37.26)	1.01 (0.15–6.95)	1.18 (0.22–6.37)
Rest of Europe, North America and Oceania	2.86 (0.46–17.83)	0.74 (0.12–4.43)	1.94 (0.57–6.65)	4.22 (0.87–20.40)	**6.36 (1.42–28.51)***	1.38 (0.49–3.85)
Middle East and North Africa	0.54 (0.09–3.41)	1.46 (0.15–14.60)	1.98 (0.56–6.97)	0.61 (0.10–3.77)	0.87 (0.17–4.44)	1.39 (0.45–4.29)
Rest of Africa	**8.29 (1.76–39.01)****	**15.06 (1.19–190.10)***	**6.13 (1.73–21.76)****	**6.63 (1.70–25.86)****	**15.50 (3.44–69.76)*****	0.55 (0.13–2.27)
Asia	0.40 (0.04–3.93)	0.40 (0.09–1.82)	1.56 (0.36–6.71)	2.49 (0.30–20.43)	0.13 (0.01–1.18)	1.08 (0.37–3.14)

[1]Adjusted for age and sex; the reference group are those who percieved they did not receive fully adequate information on COVID-19 and related restrictive measures.

*p<0.05; **p<0.01; ***p<0.001

For peer review only - http://bmjopen.bmj.com/site/about/guidelines.xhtml

DISCUSSION

Perceived access to information and self-reported adherence to preventive measures were high both in the migrant origin and the general population. In the general population, higher education, whereas in the migrant origin population, longer length of residence and excellent Finnish/Swedish language skills, were associated with higher perceived access to information. In the general population, being a woman, older age, and higher education were positively associated with adherence to preventive measures. While women reported higher adherence to preventive measures also in the migrant origin population, the association of other sociodemographic characteristics was less consistent than in the general population. Perceived sufficient access to information was associated with keeping a safety distance to others in the migrant origin population. In the analyses by region of origin, perceived access to information was associated with adherence to all preventive measures except for downloading the Koronavilkku contact tracing app among persons born in Africa ~~(excl. North Africa)~~.

Despite overall high perceived access to information, migrant origin populations reported they would have needed more information on Covid-19 more frequently than the general population. Furthermore, some regional group differences were observed, which may be attributable to the observed lower Finnish/Swedish language proficiency and shorter length of residence in some groups. Findings on the association of proficiency in Finnish/Swedish with perceived access to information are consistent with findings of a qualitative study conducted among Russian, Arabic, and Somali-speaking populations at the start of the Covid-19 pandemic in Finland[8]. In the current study, perceived access to information was poorer in those who spoke Finnish/Swedish at intermediate level. This may reflect the complexity of crisis communications. Those who do not have sufficient Finnish/Swedish skills, and for whom official information on the Finnish context was not available in own language may be at a particular risk of not receiving sufficient information. While majority (91%) of migrant origin persons reported using Finnish media, use of international media (85%) and social media (82%), which may not necessarily convey accurate information of relevance to the Finnish context, were also frequently reported[16]. Future studies should explore the association of used sources with perceived access to information.

The observed association between length of residence with perceived access to information may be related to higher likelihood of attaining better Finnish/Swedish language proficiency with longer length of residence in Finland. It may also reflect accumulation of better general knowledge on where to source relevant information. The observed association of higher education with higher perceived access to information among the general population in this study is consistent with previous studies conducted among general populations[24]. Persons with higher education may be more skilled in seeking health-related information and may have better health literacy[25].

Self-reported adherence to preventive measures was overall high both among the general and migrant origin population in the current study, which is consistent with findings from Norway[16] and Switzerland[26], but contradictory with findings from France[27]. Partially contradictory findings may be attributable to the fact that the study in France focused specifically on a disadvantaged migrant origin group from one specific region of origin, whereas the other studies included more heterogenous populations. In the current study, some differences in adherence to preventive measures were observed by region of origin. Lower adherence to preventive measures among persons born in Russia/FSU and Estonia compared with other groups may partially reflect behavioural patterns and official recommendations in the country of origin of participants. In Russia and Estonia, adherence to preventive measures has been

For peer review only - http://bmjopen.bmj.com/site/about/guidelines.xhtml

reportedly influenced by the authorities' ambivalent and dismissive attitudes and the citizens' perceived lack of support[28-29]. In contrast, persons born in the Middle East and North Africa and the rest of Africa reported very high adherence to all preventive measures in this study, except for downloading the contact tracing app. It is possible that these groups overreported adherence to preventive measures due to social desirability. However, persons originating from the Middle East and Africa also had the highest incidence of Covid-19 in Finland[30], therefore it is possible that these groups were more motivated to follow recommendations due to higher perceived risk of Covid-19 infection.

Women reported adherence to preventive measures more often than men both in the general and migrant origin populations, which is in consistency with previous studies[24]. Also in other areas of health and health-related behaviours, women generally have healthier habits than men[18, 31]. Findings of a positive association of older age with improved hand and coughing hygiene, use of face mask, and keeping a safety distance to others in the general population is also in consistency with previous studies[24, 32]. In contrast, among the migrant origin population, older age was associated with being less likely to stay at home with flu symptoms. The reasons for this should be explored in future studies. Working conditions may also be associated with adherence to preventive measures. Less than a third of participants in the MigCOVID Survey who were working or training reported they were able to work remotely, 72% were able to take care of their hand hygiene at work, 56% were able to keep a safety distance to others[16]. Future studies should also explore the association of working conditions, such as ability to work remotely and avoid close contact with others, as well as the association of job security, with adherence to preventive measures.

Migrant origin populations reported downloading the contact tracing app less frequently than the general population, and the inverse association of older age with downloading the app. This is in consistency previous studies and has been suggested to be related to lower digital competency among older adults[33-34]. Some of the participants indicated distrust towards authorities as a reason for not downloading the app, which is a concern raised among migrant origin populations also in previous studies[35]. Higher education was associated with higher likelihood of downloading the contact tracing app both among the general and migrant origin populations. The observed differences in how sociodemographic characteristics are associated with adherence to preventive measures in migrant origin compared with the general population point to a need for tailoring population-level measures for them to be effective in influencing behaviours of diverse population groups.

In the current study, little association between perceived access to information and adherence to preventive measures was observed. Both perceived access to information and self-reported adherence to preventive measures were high, which may explain lack of the observed associations. However, when examined by region of origin, the association of adequate access to information and adherence to preventive measures was particularly observed among persons born in Africa ~~(excl. North Africa)~~. A large proportion of persons belonging to this group were of Somali origin, which is a very close-knit community. Over the course of Covid-19, Somali origin community representatives and non-governmental associations were very active in distributing information within the community. Also the Somali-speaking TV channel was active in disseminating information on Covid-19. Other possible reasons for the observed association should be explored in further studies. During the Covid-19 pandemic in Finland, social listening was introduced to gather signals from diverse population groups on their perceptions and needs related to the crisis[36]. Multilingual crisis communications were developed in close collaboration with regional health and welfare professionals, key community representatives and migrant origin populations. In addition to these, Covid-19 incidence was regularly followed and the MigCOVID Survey, which constitutes the basis for

For peer review only - http://bmjopen.bmj.com/site/about/guidelines.xhtml

this study, was launched to gather population-based data on experiences during Covid-19 among migrant origin populations. When further developing future crisis preparedness, it may be worthwhile to consider use of additional systematic theory-driven tools, such as the framework for evaluating the effectiveness and sustainability of behaviourally and culturally informed interventions in complex settings, recently launched by the World Health Organisation[37].

Significant strengths of the current study are its population-based study design, representative sample, and availability of the general population reference group. The survey was available in 18 of the most commonly spoken languages in Finland, reducing language barriers for majority of the participants. Participation barriers were further reduced through provision of the questionnaire in both electronic and paper-based format, as well as by providing an alternative to respond via a telephone interview. Some limitations also need to be addressed. Self-reported access to information does not provide information on the quality of the used sources or whether the information was understood and applied as intended. There may be some report bias in the self-reported adherence to preventive measures due to social desirability. It may also be that respondents reported adherence to preventive measures, even though they may have not adhered to them in all situations. Measured access to information, that may have reduced potential response bias was not included in the current survey due to restrictions in the length of the questionnaire. However, when possible, researches designing similar surveys in the future are encouraged to consider also including measured access to information. While considerable measures were taken to increase participation rate, the 18 languages in which the questionnaire was available did not exhaustively cover all of the different languages spoken by the participants. Those who did not participate may have had poorer literacy skills, poorer command of Finnish/Swedish, and may have been likely to speak the languages in which the survey was provided. The languages selected in the survey covered the most commonly spoken languages in Finland. These broadly speaking (but not exactly) corresponded to the languages in which multilingual communications were produced in Finland. Therefore, it is possible that those who did not participate may have been at a greater risk of not receiving sufficient information. Furthermore, those who did not participate may have been less interested in their health and hence less likely to seek information or adhere to preventive measures. Non-response bias was addressed in this study through application of inverse probability weights[23]. These were based on register information on sociodemographics, and for the participants in the FinMonik sample, also earlier survey data on socioeconomic position and type of municipality of residence[4]. While the sample size and participation rate were substantial compared with other surveys conducted among migrant origin population during Covid-19, not all of the analyses could have been performed by region of origin.

In conclusion, this study provided evidence on importance of multilingual communications in securing equity in access to information among migrant origin populations in crisis situations. While policy and practice acknowledging the increasing cultural and linguistic diversity of populations has been evidently lacking prior to the pandemic, a number of countries have developed measures to improve access to up-to-date crisis communications in multiple languages during Covid-19. These measures need to be carefully documented and synthesised to identify best practices and to encourage mutual learning, as well as to facilitate better preparedness in future crises. Population diversity is a global trend that is accelerating rapidly. Therefore, it is imperative that multilingual and crisis communications are included in crisis preparedness plans. Countries must be ready to produce crisis communications in multiple languages without a significant time lag from the onset of a crisis to ensure adequate access to information among diverse population groups. Findings on differences in sociodemographic characteristics associated with access to information and adherence to preventive measures across migrant origin populations and also in

For peer review only - http://bmjopen.bmj.com/site/about/guidelines.xhtml

comparison with the general population highlight the need for acknowledging not only population's linguistic, but also cultural diversity. It is imperative to apply comprehensive theory-driven monitoring and impact assessment measures to analyse the needs, perceptions, and attitudes of diverse population groups during crisis situations to gain insights on drivers of behaviours, as well as to identify most effective strategies for influencing behaviors.

For peer review only

For peer review only - http://bmjopen.bmj.com/site/about/guidelines.xhtml

Author contributions: NS conceptualised the paper. NS drafted the manuscript with assistance of TP. TP conducted the literature review. EL conducted the statistical analyses. PK and AC have provided their critical comments for developing the manuscript. All authors have participated in interpreting the results and reviewing drafts. All authors have read and approved the manuscript sent for publication.

Funding: The MigCOVID Survey and the FinHealth 2017 follow-up Study were funded from the Finnish parliament's additional budget allocated for COVID-19 research (grant number N/A). NS and EL received funding from the Coping of disabled persons and persons of migrant origin in exceptional and crisis situations – Building the future based on experiences during Covid-19 (Building the Future) project supported by the European Social Fund (grant number S22389). TP was funded by the Impact of coronavirus epidemic on wellbeing among foreign born population (MigCOVID) Survey. The funding bodies had no role in study design; in the collection, analysis and interpretation of data; in the writing of the report; or in the decision to submit the article for publication.

Acknowledgements: The authors would like to acknowledge all of the participants in the MigCOVID Survey and the Health 2017 Study, fieldwork personnel, as well as the experts involved in planning and data management of the studies.

Data availability statement: Data are available upon reasonable request.

Competing interest statement: There are no competing interests for any author.

Ethics approval: All participants were made aware of the voluntary nature of participation in the surveys and that by participating, they permitted the use of their personal information according to the data protection notification on handling personal data. The MigCOVID Survey received ethical permission from the Ethical Committee of THL (THL/4061/6.02.01/2020). The FinHealth 2017 follow-up Study received ethical permission from the Ethics Committee II of the Helsinki and Uusimaa hospital region (HUS/2391/2020).

For peer review only

For peer review only - http://bmjopen.bmj.com/site/about/guidelines.xhtml

1 Diaz E, Norredam M, Aradhya S, et al. Situational brief: Migration and COVID-19 in Scandinavian countries, *Econ Geogr* 2021.

2 Li SL, Pereira RHM, Prete CA, et al. Higher risk of death from COVID-19 in low-income and non-White populations of Sao Paulo, Brazil, *BMJ Glob Health* 2021;6:10.1136/bmjgh-004959 doi:e004959 [pii] [published Online First: April 01].

3 Hayward SE, Deal A, Cheng C, et al. Clinical outcomes and risk factors for COVID-19 among migrant populations in high-income countries: A systematic review, *J Migr Health* 2021;3:100041 doi:10.1016/j.jmh.2021.100041 [doi].

4 Skogberg N, Koponen P, Lilja E, et al. Impact of Covid-19 on the health and wellbeing of persons who migrated to Finland: The MigCOVID Survey 2020-2021, 2021.

5 Schmidt H, Wild E, Schreyögg J. Explaining variation in health information seeking behaviour – Insights from a multilingual survey, *Health policy (Amsterdam)* 2021;125:618-26 doi:10.1016/j.healthpol.2021.01.008 [published Online First: May].

6 Massaro M, Tamburro P, La Torre M, et al. Non-pharmaceutical Interventions and the Infodemic on Twitter: Lessons Learned from Italy during the Covid-19 Pandemic, *J Med Syst* 2021;45:50-7 doi:10.1007/s10916-021-01726-7 [doi] [published Online First: March 06].

7 Brønholt RLL, Langer Primdahl N, Jensen AMB, et al. "I Just Want Some Clear Answers": Challenges and Tactics Adopted by Migrants in Denmark When Accessing Health Risk Information about COVID-19, *Int J Environ Res Public Health* 2021;18:8932 doi:10.3390/ijerph18178932 [published Online First: Aug 25,].

8 Finell E, Tiilikainen M, Jasinskaja-Lahti I, et al. Lived Experience Related to the COVID-19 Pandemic among Arabic-, Russian- and Somali-Speaking Migrants in Finland, *Int J Environ Res Public Health* 2021;18:10.3390/ijerph18052601 doi:2601 [pii] [published Online First: March 05].

9 Knights F, Carter J, Deal A, et al. Impact of COVID-19 on migrants' access to primary care and implications for vaccine roll-out: a national qualitative study, *Br J Gen Pract* 2021;71:e583-95 doi:10.3399/BJGP.2021.0028 [doi] [published Online First: July 29].

10 Deal A, Hayward SE, Huda M, et al. Strategies and action points to ensure equitable uptake of COVID-19 vaccinations: A national qualitative interview study to explore the views of undocumented migrants, asylum seekers, and refugees, *J Migr Health* 2021;4:100050 doi:10.1016/j.jmh.2021.100050 [doi].

11 Wang C, Tian Q, Zhao P, et al. Disease knowledge and attitudes during the COVID-19 epidemic among international migrants in China: a national cross-sectional study, *Int J Biol Sci* 2020;16:2895-905 doi:10.7150/ijbs.47075 [doi] [published Online First: September 04].

12 Valeriani G, Vukovic IS, Mollica R. Unconventional Answers to Unprecedented Challenges: The Swedish Experience During the COVID-19 Outbreak, *J Prev Med Public Health* 2020;53:233-5.

13 Nezafat Maldonado BM, Collins J, Blundell HJ, et al. Engaging the vulnerable: A rapid review of public health communication aimed at migrants during the COVID-19 pandemic in Europe, *J Migr Health* 2020;1:100004 doi:10.1016/j.jmh.2020.100004 [doi].

14 Skogberg N, Hussein I, Castaneda A. Impact of Covid-19 human mobility restrictions on migrant origin population in Finland, *in book: COVID-19 and Migration: Understanding Pandemic and Human Mobility.Published* 2020;11.

15 Statistics Finland. Immigrants in the population. Official statistics of Finland, 2022. Accessed February 1st, 2023 from: https://www.stat.fi/tup/maahanmuutto/maahanmuuttajat-vaestossa_en.html.

For peer review only - http://bmjopen.bmj.com/site/about/guidelines.xhtml

16 Skogberg N, Koponen P, Lilja E, et al. Access to information, preventive measures and working conditions during the coronavirus epidemic: Findings of the population-based MigCOVID Survey among persons who have migrated to Finland, 2021.

17 Madar AA, Benavente P, Czapka E, et al. COVID-19: information access, trust and adherence to health advice among migrants in Norway, *Archives of public health = Archives belges de santé publique* 2022;80:15 doi:10.1186/s13690-021-00764-4 [published Online First: Jan 4,].

18 Kuusio H, Seppänen A, Jokela S, et al. Ulkomaalaistaustaisten terveys ja hyvinvointi Suomessa: FinMonik-tutkimus 2018-2019, 2020.

19 Borodulin K, Sääksjärvi K. FinHealth 2017 Study: Methods, 2019. Finnish Institute for health and Welfare, THL; Helsinki.

20 United Nations. Standard country or area codes for statistical use (M49). 2021.

21 Graubard BI, Korn EL. Predictive margins with survey data, *Biometrics* 1999;55:652-9.

22 Lehtonen R, Pahkinen E. Practical methods for design and analysis of complex surveys: John Wiley & Sons 2004.

23 Robins JM, Rotnitzky A, Zhao LP. Estimation of regression coefficients when some regressors are not always observed, *J Am Stat Assoc* 1994;89:846-66.

24 Varghese NE, Sabat I, Neumann-Böhme S, et al. Risk communication during COVID-19: A descriptive study on familiarity with, adherence to and trust in the WHO preventive measures, *PloS one* 2021;16:e0250872.

25 Demirci Ş, Uğurluoğlu Ö, Konca M, et al. Socio-demographic characteristics affect health information seeking on the Internet in Turkey, *Health Info Libr J* 2021;38:304-12.

26 Nivette A, Ribeaud D, Murray A, et al. Non-compliance with COVID-19-related public health measures among young adults in Switzerland: Insights from a longitudinal cohort study, *Social science & medicine (1982)* 2021;268:113370 doi:10.1016/j.socscimed.2020.113370 [published Online First: Jan].

27 Gosselin A, Melchior M, Carillon S, et al. Deterioration of mental health and insufficient Covid-19 information among disadvantaged immigrants in the greater Paris area, *J Psychosom Res* 2021;146:110504 doi:S0022-3999(21)00149-5 [pii] [published Online First: July 01].

28 Margraf J, Brailovskaia J, Schneider S. Adherence to behavioral Covid-19 mitigation measures strongly predicts mortality, *PloS one* 2021;16:e0249392 doi:10.1371/journal.pone.0249392.

29 Margraf J, Brailovskaia J, Schneider S. Behavioral measures to fight COVID-19: An 8-country study of perceived usefulness, adherence and their predictors, *Plos one* 2020;15:e0243523.

30 Holmberg V, Salmi H, Kattainen S, et al. Association between first language and SARS-CoV-2 infection rates, hospitalization, intensive care admissions and death in Finland: a population-based observational cohort study, *Clin Microbiol Infect* 2022;28:107-13 doi:10.1016/j.cmi.2021.08.022 [published Online First: Jan].

31 Koponen P, Borodulin K, Lundqvist A, et al. Terveys, toimintakyky ja hyvinvointi Suomessa: FinTerveys 2017 -tutkimus. Terveyden ja hyvinvoinnin laitos, raportti 4/2018.

32 Bearth A, Luchsinger L, Siegrist M. Reactions of older Swiss adults to the COVID-19 pandemic: A longitudinal survey on the acceptance of and adherence to public health measures, *Soc Sci Med* 2021;280:114039.

For peer review only - http://bmjopen.bmj.com/site/about/guidelines.xhtml

33 Dowthwaite L, Fischer J, Vallejos EP, et al. Public Adoption of and Trust in the NHS COVID-19 Contact Tracing App in the United Kingdom: Quantitative Online Survey Study, *J Med Internet Res* 2021;23:e29085.

34 Skarpa PE, Garoufallou E. Information seeking behavior and COVID-19 pandemic: A snapshot of young, middle aged and senior individuals in Greece, *Int J Med Inf* 2021;150:104465.

35 Abba-Aji M, Stuckler D, Galea S, et al. Ethnic/racial minorities' and migrants' access to COVID-19 vaccines: A systematic review of barriers and facilitators. *J Migr Health* 2022:100086.

36 Lohiniva A, Sibenberg K, Austero S, et al. Social Listening to Enhance Access to Appropriate Pandemic Information Among Culturally Diverse Populations: Case Study From Finland *JMIR Infodemiology* 2022;2(2):e38343 doi: 10.2196/38343

37 World Health Organization. Guide to evaluating behaviourally and culturally informed health interventions in complex settings. 2022. https://www.who.int/europe/publications/i/item/WHO-EURO-2022-6045-45810-65956

For peer review only

STROBE 2007 (v4) Statement—Checklist of items that should be included in reports of *cross-sectional studies*

Section/Topic	Item #	Recommendation	Reported on page #
**Title and abstract**	1	(*a*) Indicate the study's design with a commonly used term in the title or the abstract	1,2
		(*b*) Provide in the abstract an informative and balanced summary of what was done and what was found	2
**Introduction**			
Background/rationale	2	Explain the scientific background and rationale for the investigation being reported	4
Objectives	3	State specific objectives, including any prespecified hypotheses	4
**Methods**			
Study design	4	Present key elements of study design early in the paper	5
Setting	5	Describe the setting, locations, and relevant dates, including periods of recruitment, exposure, follow-up, and data collection	5
Participants	6	(*a*) Give the eligibility criteria, and the sources and methods of selection of participants	5
Variables	7	Clearly define all outcomes, exposures, predictors, potential confounders, and effect modifiers. Give diagnostic criteria, if applicable	5,6
Data sources/ measurement	8*	For each variable of interest, give sources of data and details of methods of assessment (measurement). Describe comparability of assessment methods if there is more than one group	5,6
Bias	9	Describe any efforts to address potential sources of bias	*6*
Study size	10	Explain how the study size was arrived at	5
Quantitative variables	11	Explain how quantitative variables were handled in the analyses. If applicable, describe which groupings were chosen and why	5,6
Statistical methods	12	(*a*) Describe all statistical methods, including those used to control for confounding	6
		(*b*) Describe any methods used to examine subgroups and interactions	6
		(*c*) Explain how missing data were addressed	6
		(*d*) If applicable, describe analytical methods taking account of sampling strategy	6
		(*e*) Describe any sensitivity analyses	-
**Results**			

For peer review only

Participants	13*	(a) Report numbers of individuals at each stage of study—eg numbers potentially eligible, examined for eligibility, confirmed eligible, included in the study, completing follow-up, and analysed	5
		(b) Give reasons for non-participation at each stage	5
		(c) Consider use of a flow diagram	-
Descriptive data	14*	(a) Give characteristics of study participants (eg demographic, clinical, social) and information on exposures and potential confounders	16
		(b) Indicate number of participants with missing data for each variable of interest	16-19
Outcome data	15*	Report numbers of outcome events or summary measures	16-19
Main results	16	(*a*) Give unadjusted estimates and, if applicable, confounder-adjusted estimates and their precision (eg, 95% confidence interval). Make clear which confounders were adjusted for and why they were included	16-19
		(*b*) Report category boundaries when continuous variables were categorized	-
		(*c*) If relevant, consider translating estimates of relative risk into absolute risk for a meaningful time period	-
Other analyses	17	Report other analyses done—eg analyses of subgroups and interactions, and sensitivity analyses	8
**Discussion**			
Key results	18	Summarise key results with reference to study objectives	8
Limitations	19	Discuss limitations of the study, taking into account sources of potential bias or imprecision. Discuss both direction and magnitude of any potential bias	9
Interpretation	20	Give a cautious overall interpretation of results considering objectives, limitations, multiplicity of analyses, results from similar studies, and other relevant evidence	8,9
Generalisability	21	Discuss the generalisability (external validity) of the study results	10
**Other information**			
Funding	22	Give the source of funding and the role of the funders for the present study and, if applicable, for the original study on which the present article is based	11

*Give information separately for cases and controls in case-control studies and, if applicable, for exposed and unexposed groups in cohort and cross-sectional studies.

Note: An Explanation and Elaboration article discusses each checklist item and gives methodological background and published examples of transparent reporting. The STROBE checklist is best used in conjunction with this article (freely available on the Web sites of PLoS Medicine at http://www.plosmedicine.org/, Annals of Internal Medicine at http://www.annals.org/, and Epidemiology at http://www.epidem.com/). Information on the STROBE Initiative is available at www.strobe-statement.org.

For peer review only

BMJ Open

The association of sociodemographic characteristics with self-perceived access to Covid-19 information and adherence to preventive measures among migrant origin and general populations in Finland: a cross-sectional study

Journal:	*BMJ Open*
Manuscript ID	bmjopen-2022-069192.R2
Article Type:	Original research
Date Submitted by the Author:	15-Feb-2023
Complete List of Authors:	Skogberg, N. ; National Institute for Health and Welfare, Helsinki, Finland, Welfare<br>Prinkey, Tyler; Finnish Institute for Health and Welfare<br>Lilja, Eero; National Institute for Health and Welfare, Welfare<br>Koponen, Päivikki; National Institute for Health and Welfare, Public Health Solutions<br>Castaneda, Anu; Terveyden ja hyvinvoinnin laitos,
**Primary Subject Heading**:	Public health
Secondary Subject Heading:	Communication, Epidemiology, Evidence based practice, Global health, Health policy
Keywords:	COVID-19, Public health < INFECTIOUS DISEASES, PUBLIC HEALTH

SCHOLARONE™
Manuscripts

I, the Submitting Author has the right to grant and does grant on behalf of all authors of the Work (as defined in the below author licence), an exclusive licence and/or a non-exclusive licence for contributions from authors who are: i) UK Crown employees; ii) where BMJ has agreed a CC-BY licence shall apply, and/or iii) in accordance with the terms applicable for US Federal Government officers or employees acting as part of their official duties; on a worldwide, perpetual, irrevocable, royalty-free basis to BMJ Publishing Group Ltd ("BMJ") its licensees and where the relevant Journal is co-owned by BMJ to the co-owners of the Journal, to publish the Work in this journal and any other BMJ products and to exploit all rights, as set out in our licence.

The Submitting Author accepts and understands that any supply made under these terms is made by BMJ to the Submitting Author unless you are acting as an employee on behalf of your employer or a postgraduate student of an affiliated institution which is paying any applicable article publishing charge ("APC") for Open Access articles. Where the Submitting Author wishes to make the Work available on an Open Access basis (and intends to pay the relevant APC), the terms of reuse of such Open Access shall be governed by a Creative Commons licence – details of these licences and which Creative Commons licence will apply to this Work are set out in our licence referred to above.

Other than as permitted in any relevant BMJ Author's Self Archiving Policies, I confirm this Work has not been accepted for publication elsewhere, is not being considered for publication elsewhere and does not duplicate material already published. I confirm all authors consent to publication of this Work and authorise the granting of this licence.

For peer review only

Title: The association of sociodemographic characteristics with self-perceived access to Covid-19 information and adherence to preventive measures among migrant origin and general populations in Finland: a cross-sectional study

Natalia Skogberg*[a], Tyler Prinkey[a], Eero Lilja[a], Päivikki Koponen[a], Anu E Castaneda[a]. [The surname of each author is underlined]

*Corresponding author; natalia.skogberg@thl.fi; ORCHID: 0000-0002-8829-4159. Address: Finnish Institute for Health and Welfare, Mannerheimintie 166, P.O. box 27, 00271 Helsinki. Tel. +358 29 524 7916.

[a]Department of Public Health and Welfare, Finnish institute for Health and Welfare, Helsinki, Finland.

word count: 4597

tables: 5

For peer review only

Abstract

Objectives: This study examines how access to Covid-19 information and adherence to preventive measures varies by sociodemographic characteristics, and whether the associations differ among the migrant origin and the general Finnish population. Additionally, the association of perceived access to information with adherence to preventive measures is examined.

Design: Cross-sectional, population-based random sample.

Background: Equity in access to information is crucial for securing individual wellbeing and successful management of a crisis at population level.

Setting: Persons who have a residence permit in Finland.

Participants: Migrant origin population constitutes of persons born abroad aged 21 to 66 years, who took part in the MigCOVID Survey conducted 10/2020–2/2021 (n=3611). Participants in the Health 2017 follow-up survey conducted within the same time frame, representing the general Finnish population consitituted the reference group (n=3 490).

Outcome measures: Self-perceived access to Covid-19 information, adherence to preventive measures.

Results: Self-perceived access to information and adherence to preventive measures was overall high both among the migrant origin and the general population. Perceived adequate access to information was associated with living in Finland for 12 years or longer (OR 1.94, 95%CI 1.05-3.57) and excellent Finnish/Swedish language skills (OR 2.71, 95% CI 1.62-4.53) among the migrant origin population and with higher education (OR 3.56, 95% CI 1.49-8.55 for tertiary and OR 2.87, 95%CI 1.25-6.59 for secondary) among the general population. The association between examined sociodemographic characteristics with adherence to preventive measures varied by study group.

Conclusions: Findings on the association of perceived access to information with language proficiency in official languages highlight the need for rapid multilingual and simple language crisis communications. Findings also suggest that crisis communications and measures designed to influence health behaviours at population level may not be directly transferable if the aim is to influence health behaviours also among ethnically and culturally diverse populations.

key words: migrant, Covid-19, communication, MigCOVID, prevention

For peer review only

Strengths and limitations of the study

- Significant strengths of the current study are its population-based study design, representative sample, and availability of the general population reference group.
- Barriers to participation were reduced by making the survey material available in 18 languages both in electronic and paper-based format, as well as by providing with the option to respond via a telephone interview conducted by multilingual interviewers.
- One limitation is that self-reported access to information does not provide information on the quality of the used sources or whether the information was understood and applied as intended.
- There may be some report bias in the self-reported adherence to preventive measures due to social desirability. It may also be that respondents reported adherence to preventive measures, even though they may have not adhered to them in all situations.

For peer review only

For peer review only - http://bmjopen.bmj.com/site/about/guidelines.xhtml

BACKGROUND

Covid-19 exposed the weakest spots in crisis preparedness, highlighting the evident lack of policies and practices that acknowledge increasing globalization and population cultural diversity[1]. Vulnerabilities of migrant origin populations were reflected in significantly higher incidence of Covid-19 infections, higher risk for hospitalizations, mortality rates, as well as higher economic, social, and psychological impact of the pandemic compared with general populations[2-4]. Information guidance was crucial in management of the pandemic, however crisis communications were challenged by ever-evolving information and changes in official guidelines[5- 6]. From the onset of the pandemic, significant concerns were raised regarding equity in access to information among migrant origin populations. Difficulties in accessing up-to-date and reliable information occurred due to language barriers, lack of trust in the health system, and gaps in health and digital literacy[7-9]. Inequities in access to information may lead to stress, lower awareness, and hence also lower adherence to preventive measures[7,10-11]. In addition to impact on individual-level coping in a crisis, these may negatively impact overall crisis management and hence may jeopardise societal security.

While multilingual communications and community outreach programmes were launched in a number of countries to improve equity in access to information across different population groups[12], practices across countries varied. During the first half year of the pandemic, only half of European countries had information on Covid-19 available in at least one foreign language, and only several countries had information in multiple foreign languages[13]. Significant efforts have been placed to develop multilingual Covid-19 communications also in Finland[14]. Finland is a country in North of Europe, with the population of 5.6 million. Migration to Finland rapidly accelerated over the past decades, from 0.5% of the total population in the 1990 to 8% in 2021[15]. In 2021, the largest migrant origin populations in Finland were persons born in the former Soviet Union, Estonia, Russian Federation, Sweden, Irak, China, and Somalia. A few weeks into the pandemic, the Finnish Institute for Health and Welfare (THL) introduced information on Covid-19 in 16 foreign languages. Language selection was later expanded by several additional languages. In addition to THL that produced crisis communications at the national level, regional health authorities and non-governmental organisations also produced multilingual communications. It was well acknowledged that simply publishing multilingual information is not sufficient, and particular efforts were taken to ensure effective information dissemination to the target group. Dissemination of national-level Covid-19 communications was implemented by THL's Multilingual and multichannel communications task force in close collaboration with regional health and social welfare professionals, non-governmental organisations, religious community leaders, and other key community representatives[16].

Because multilingual Covid-19 comminications were available with a slight delay, migrant origin populations in Finland initially perceived they did not receive sufficient information, but the situation improved over the course of the pandemic[8]. To date, studies exploring perceived access to information and adherence to preventive measures among migrant origin populations have been generally based on qualitative and non-representative samples[7-9, 17]. The general population reference group is rarely available, which makes contextualisation of the findings challenging. There is also limited information on how access to information and adherence to preventive measures has varied by sociodemographic characteristics, and whether these differed between migrant origin and the general population. Furthermore, there is limited information on how perceived access to information has impacted behaviours in different population groups. This information is needed for evaluation of crisis response measures and for identifying groups at highest risk of vulnerabilities in crisis situations. These all significantly contribute to development of preparedness in future crises. The aim of the current study is to fill this gap in knowledge by using

For peer review only - http://bmjopen.bmj.com/site/about/guidelines.xhtml

representative population-based data to examine how access to Covid-19 information and adherence to preventive measures varied by sociodemographic characteristics, and whether these differed between the migrant origin and the general Finnish populations. Additionally, the association of access to information with adherence to preventive measures is examined.

METHODS

The present study is based on the Impact of the Coronavirus on the Wellbeing of the Foreign Born Population (MigCOVID) Survey. The survey was conducted between October 2020 and February 2021, coinciding with the second wave of the Covid-19 pandemic in Finland. A more detailed description of the survey can be found elsewhere[4]. Briefly, the MigCOVID Survey was a 2-year follow-up study to the Survey on Wellbeing among Foreign Born Population (FinMonik) [18]. The original stratified random sample was drawn from the Finnish Population Register using the following selection criteria: age 18-64 years, both the person belonging to the sample and their parents were born outside of Finland, and residence in Finland for at least one year. Following exclusion of persons declining further contact during the FinMonik Survey and who no longer lived in Finland, the updated sample of the MigCOVID Survey comprised of 5 269 persons aged 20 to 66 years. In addition to including participants in the FinMonik Survey, a supplementary sample of 982 persons of Somali origin was drawn using simple random sampling and following the same selection criteria as the original sample. Following exclusion of overcoverage (n=112), the final sample of the MigCOVID Survey constituted of 6 139 persons. Altogether 3668 persons took part in the MigCOVID Survey, with a participation rate of 60%. Since data for the general population reference group was available only for persons aged 21 years and older, n=57 persons below 21 years were excluded from the MigCOVID participants, resulting in use of data of n=3611 participants in the MigCOVID Survey in the current study.

Invitation letters were sent by mail with personal login information to the electronic questionnaire. Data collection was supplemented with mailed paper questionnaires and telephone interviews. Invitation letters, information about the survey, and questionnaires were available in 18 languages. Invitation letters, and paper questionnaires were sent out to the participants in Finnish or Swedish, depending on which of these was marked as the preferred language of communication in the Finnish Population Register, and in their official mother tongue as marked in register. If the questionnaire was not available in the register-based mother tongue of the participant, the post was sent in Finnish/Swedish only. Participants could themselves select the language in which they filled out the electronic questionnaire out of the available 18 languages. Telephone interviews were carried out by trained multilingual fieldwork personnel in 12 languages.

A reference group was formed from a sub-population of the FinHealth 2017 follow-up Study of corresponding age to the participants in the MigCOVID Survey. As the youngest participants in the FinHealth 2017 follow-up Study were 21 years, the reference group of this study constitutes of persons aged 21 to 66 years (n=3490 participated, participation rate 51%). The FinHealth 2017 Study had a nationally representative, two-stage stratified cluster sample of adults living in Finland[19]. The study was conducted within the same time frame and following a similar protocol to the MigCOVID Survey.

Patient and public involvement in research

This study does not involve patients. The public were not involved in the planning or design of the research. Data collection was implemented by trained multilingual fieldwork personnel. Findings have been disseminated to the public in multiple languages through the webpages of the survey and social media.

For peer review only - http://bmjopen.bmj.com/site/about/guidelines.xhtml

Additionally, group discussions with representatives of different migrant origin populations have been conducted to disseminate and to hear feedback on the findings.

Variable definitions

Information on date of birth, age, sex, and length of residence in Finland was obtained from the Finnish Population Register. The MigCOVID Survey participants were grouped into six regions of origin based on their country of birth following a slightly modified United Nations area code grouping[20]: Russia or the former Soviet Union (FSU); Estonia; the rest of Europe, North America, and Oceania; the Middle East and North Africa; the rest of Africa (later in the text as Africa); Asia or other (later in the text as Asia). In the latter group, "other" constituted of persons born in Latin America (n=96). Information on highest completed level of education in Finland or abroad, economic activity, and Finnish/Swedish language skills (official languages of Finland) was gathered as self-report measures during the survey. Categorization of the included sociodemographic characteristics can be found in Table 1.

Self-perceived access to information was assessed in both studies with the question: Have your received adequate information on how to avoid getting infected with the coronavirus and how to prevent it from spreading? The answer options were: I have not received any information or the information I have received has been completely inadequate; I have received information, but I would have needed more; or I have received adequate information. The first two answer options were grouped.

Adherence to preventive measures was based on the measures recommended by THL at the point of finalization of the questionnaire in September 2020. Participants were asked: Which measures have you taken to avoid getting infected with the coronavirus and prevent it from spreading: I wash my hands more frequently; I use sanitisers more frequently; I take care of hygiene when coughing (e.g. coughing into a disposable tissue, not coughing into hands; I stay at home if I have flu symptoms (e.g. cough, cold symptoms or sore throat); I wear a single use mask or cloth mask during free time (when it is not possible to avoid contact with other people); I keep a safe distance to other people outside of the home; I do not shake hands with the people I meet. The answer options were: Yes, I follow the instruction/ recommendation, and I do not follow the instruction/recommendation. The questions on washing hands, use of sanitisers and coughing hygiene were grouped into a joint variable "improved hand and coughing hygiene". Answers were dichotomized as "yes" (yes to all three) and "no" (no to at least one measure).

Participants were also asked if they have downloaded the Koronavilkku contact tracing app. In the MigCOVID Survey, the answer options were: yes; no, because the app is not available for my phone; no, because I don't know what it is; no, because the app is not available in the languages that I speak; no, for other reasons, what? The answers were dichotomised as yes/no. The open-ended answers were analysed using thematic grouping. In the Health 2017 follow-up Study, the answer options were: yes; no, because I don't have a suitable smartphone; no for other reasons. These were also dichotomized as yes or no.

Statistical analyses

The differences between groups were tested using logistic regression. P-value based on Satterthwaite-adjusted F-value, as well as odds ratio and its 95% confidence interval were used to assess the differences. The model-adjusted proportions were calculated with predictive margins[21]. The stratification of the sample was accounted for in all analyses and finite population correction was applied[22]. The data were weighted using inverse probability weights to reduce the non-response bias and to take varying sampling

For peer review only - http://bmjopen.bmj.com/site/about/guidelines.xhtml

probabilities into account[23]. The calculation of the IPWs is described elsewhere[4, 19]. All analyses were carried out with SAS 9.4 and SUDAAN 11.0.3 statistical software.

For peer review only

For peer review only - http://bmjopen.bmj.com/site/about/guidelines.xhtml

RESULTS

Descriptive characteristics of the study participants are presented in Table 1. The proportion of men was lowest among persons born in Russia/FSU and highest among persons born in the Middle East and North Africa. Persons born in Africa were the youngest. The proportion of those with at least tertiary education was lowest among persons born in Estonia and highest among persons born in the rest of Europe, North America and Oceania. Employment was lowest among persons born in the Middle East and North Africa. Persons born in Russia/FSU had lived in Finland the longest and had the highest Finnish/Swedish language proficiency.

[Table 1 about here]

Perceived access to information and adherence to preventive measures among the study participants is presented in Table 2. Migrant origin population reported receiving fully adequate Covid-19 information less frequently than the general population. Perceived access to information was lowest among persons born in Europe (excl. Russia/FSU, Estonia), North America and Oceania, the Middle East and North Africa, and Asia.

[Table 2 about here]

Persons born in Russia/FSU and Estonia reported lower, while persons born in the Middle East and Africa reported higher adherence to preventive measures compared with the general population. Migrant origin population reported downloading the Koronavilkku contact tracing app less frequently than the general population. The most common reasons mentioned for not downloading the app were not knowing about it; the app was unnecessary; not wanting to; the app would just increase stress; mistrust towards the authorities; technical difficulties; and that the app consumes too much phone memory. (results not shown)

The association of sociodemographic characteristics with self-perceived access to information and adherence to Covid-19 preventive measures among the general population is presented in Table 3. Higher education was positively associated with self-perceived sufficient access to information. Women were more likely than men to report adhering to all other preventive measures except for keeping a safety distance to others. Older age was positively associated with improved hand hygiene and use of a face mask. Additionally, persons in the oldest age group (50-66 years) were more likely to keep a safety distance to others but less likely to download the contact tracing app compared with the youngest (21-34 years) age group. Persons with tertiary education were more likely to report the use of a face mask than persons with basic education. Higher education and employment were also associated with downloading the contact tracing app.

[Table 3 about here]

For peer review only - http://bmjopen.bmj.com/site/about/guidelines.xhtml

Table 1 Descriptive characteristics of the study participants

	General population	Born abroad total	Russia or former Soviet Union	Estonia	Rest of Europe, North America, Oceania	Middle East and North Africa	Rest of Africa	Asia
	n=3490	n=3611	n=1115	n=300	n=712	n=436	n=302	n=746
	N (%)	N (%)	N (%)	N (%)	N (%)	N (%)	N (%)	N (%)
**Men**	1604 (50.5)	1614 (51.6)	380 (39.0)	110 (46.5)	399 (64.7)	286 (68.8)	174 (54.9)	265 (42.7)
**Age**								
21–34 years	533 (29.3)	1090 (34.7)	251 (24.5)	56 (25.0)	179 (32.5)	178 (49.7)	156 (45.7)	270 (38.1)
35–49 years	1142 (33.6)	1509 (41.7)	412 (38.0)	119 (39.7)	339 (48.1)	178 (36.2)	122 (46.9)	339 (43.0)
50–66 years	1815 (37)	1012 (23.6)	452 (37.5)	125 (35.3)	194 (19.4)	80 (14.1)	24 (7.4)	137 (18.9)
**Education**								
Basic education or less	273 (6.5)	380 (13.2)	57 (6.0)	32 (16.0)	55 (11.8)	86 (16.8)	56 (21.0)	94 (14.2)
Secondary education	1764 (49.7)	1276 (46.1)	435 (56.6)	147 (59.2)	210 (33.5)	160 (50.8)	117 (42.4)	207 (36.2)
Tertiary education or higher	1412 (43.8)	1888 (40.8)	609 (37.4)	113 (24.8)	440 (54.6)	174 (32.4)	119 (36.6)	433 (49.6)
**Economic activity**								
Employed full-time/part-time	2404 (70.7)	2299 (65.7)	699 (64.8)	218 (70.9)	504 (72.5)	197 (51.6)	172 (59.9)	509 (69.7)
Unemployed, student or other	1034 (29.3)	1229 (34.3)	396 (35.2)	77 (29.1)	195 (27.5)	225 (48.4)	119 (40.1)	217 (30.3)
**Length of residence in Finland**								
3–6.99 years	N/A	793 (17.9)	163 (6.7)	32 (6.5)	134 (22.6)	183 (31.8)	46 (14.3)	235 (24.0)
7–11.99 years	N/A	1020 (28.7)	273 (18.9)	124 (43.4)	200 (28.2)	116 (30.4)	58 (21.3)	249 (31.6)
12+ years	N/A	1798 (53.4)	679 (74.3)	144 (50.1)	378 (49.2)	137 (37.9)	198 (64.3)	262 (44.3)
**Excellent Finnish/Swedish language proficiency**	N/A	1364 (35.4)	480 (43.2)	182 (52.1)	273 (32.0)	122 (29.1)	163 (50.3)	144 (17.8)

For peer review only

For peer review only - http://bmjopen.bmj.com/site/about/guidelines.xhtml

Table 2 Perceived access to information and self-reported adherence to preventive measures among the study population[1]

	General population	Born abroad total	Russia or former Soviet Union	Estonia	Rest of Europe, North America, Oceania	Middle East and North Africa	Rest of Africa	Asia
	n=3490	n=3611	n=1115	n=300	n=712	n=436	n=302	n=746
	% (95%CI)	% (95%CI)	% (95%CI)	% (95%CI)	% (95%CI)	% (95%CI)	% (95%CI)	% (95%CI)
**Access to information**								
Received adequate information	97.1 (95.7–98.0)	93.5 (92.0–94.8)***	96.1 (94.3–97.4)	97.4 (94.0–98.9)	91.4 (87.0–94.5)***	91.8 (86.6–95.1)**	93.9 (88.8–96.7)	91.4 (86.8–94.5)***
Received, but would have needed more	2.5 (1.6–3.9)	5.3 (4.2–6.7)***	2.8 (1.8–4.3)	1.9 (0.7–5.5)	6.2 (3.8–9.9)**	7.8 (4.6–13.0)**	5.4 (2.7–10.6)	7.5 (4.8–11.6)***
No information/completely inadequate	0.4 (0.2–0.8)	1.1 (0.6–2.0)***	1.1 (0.5–2.4)*	0.6 (0.2–2.4)	2.4 (0.9–6.2)**	0.4 (0.1–1.1)	0.7 (0.3–1.9)	1.1 (0.2–5.6)
**Following preventive measures**								
Staying home with flu symptoms	96.1 (94.6–97.2)	96.4 (95.1–97.4)	94.2 (91.4–96.1)	92.1 (85.2–95.9)	96.4 (92.8–98.2)	99.2 (98.1–99.7)**	99.0 (97.6–99.6)**	98.2 (95.2–99.3)
Improved hand and coughing hygiene	92.2 (90.3–93.7)	92.8 (91.0–94.2)	85.3 (81.2–88.7)***	84.4 (75.7–90.4)*	96.3 (92.7–98.1)*	95.7 (91.4–97.9)	98.7 (96.9–99.5)***	96.2 (92.4–98.1)
Use of face mask	81.9 (79.6–83.9)	81.5 (79.0–83.7)	69.5 (64.1–74.4)***	65.0 (55.5–73.5)***	84.2 (78.5–88.5)	88.9 (83.5–92.7)*	93.7 (90.4–96.0)***	89.2 (84.1–92.9)***
Keeping a safety distance	94.6 (93.0–95.8)	93.5 (91.6–95.0)	92.0 (88.5–94.6)	87.1 (78.1–92.8)**	92.6 (86.6–96.0)	96.8 (93.0–98.6)	97.3 (95.3–98.5)*	94.9 (90.2–97.4)
Avoiding shaking hands with others	98.8 (98.3–99.2)	93.2 (91.5–94.6)***	89.7 (86.2–92.3)***	84.7 (75.5–90.9)***	95.8 (92.5–97.7)***	95.4 (92.2–97.4)***	95.7 (92.5–97.6)***	96.2 (92.0–98.3)**
Downloaded Koronavilkku contact tracing app	62.4 (60.1–64.7)	44.1 (41.2–47.2)***	33.5 (28.4–39.0)***	23.1 (16.6–31.2)***	53.5 (46.6–60.3)*	51.4 (43.7–59.1)**	50.8 (40.2–61.4)*	52.8 (46.0–59.4)**

[1]Adjusted for age and sex

*p<0.05; **p<0.01; ***p<0.001, in comparison with the general population

For peer review only

For peer review only - http://bmjopen.bmj.com/site/about/guidelines.xhtml

Table 3 The association of sociodemographic characteristics with perceived adequate access to information and adherence to Covid-19 related preventive measures among the general population

	Perceived access to information	Staying home with flu symptoms	Improved hand and coughing hygiene	Use of face mask	Keeping a safety distance to others	Avoiding shaking hands with others	Downloaded Koronavilkku app
	OR (95%CI)	OR (95%CI)	OR (95%CI)	OR (95%CI)	OR (95%CI)	OR (95%CI)	OR (95%CI)
**Sex**[1]							
Men	1.00	1.00	1.00	1.00	1.00	1.00	1.00
Women	0.75 (0.36–1.55)	**2.53 (1.14–5.59)***	**2.43 (1.58–3.74)*****	**3.30 (2.49–4.38)*****	1.17 (0.68–2.02)	**2.46 (1.16–5.19)***	**1.64 (1.36–1.98)*****
**Age**[2]							
21–34 years	1.00	1.00	1.00	1.00	1.00	1.00	1.00
35–49 years	1.51 (0.59–3.89)	1.35 (0.61–3.00)	**1.92 (1.11–3.32)***	**1.50 (1.03–2.21)***	1.24 (0.65–2.39)	2.17 (0.90–5.25)	0.79 (0.58–1.07)
50–66 years	2.08 (0.80–5.36)	2.21 (0.99–4.91)	**2.08 (1.23–3.52)****	**2.05 (1.41–2.97)*****	**2.46 (1.27–4.79)****	1.96 (0.86–4.45)	**0.61 (0.45–0.81)*****
**Education**[3]							
Basic education or less	1.00	1.00	1.00	1.00	1.00	1.00	1.00
Secondary education	**2.87 (1.25–6.59)***	1.13 (0.48–2.69)	1.12 (0.37–3.36)	1.28 (0.88–1.87)	0.94 (0.43–2.04)	1.12 (0.37–3.36)	**2.13 (1.58–2.87)*****
Tertiary education or higher	**3.56 (1.49–8.55)****	1.51 (0.61–3.75)	1.19 (0.37–3.86)	**2.20 (1.41–3.44)*****	1.10 (0.49–2.47)	1.19 (0.37–3.86)	**4.48 (3.22–6.24)*****
**Economic activity**[3]							
Unemployed, student or other	1.00	1.00	1.00	1.00	1.00	1.00	1.00
Employed full-time/part-time	0.88 (0.37–2.09)	1.81 (0.86–3.83)	0.89 (0.40–1.96)	0.93 (0.66–1.30)	0.80 (0.40–1.59)	0.89 (0.40–1.96)	**0.63 (0.50–0.79)*****

[1]Adjusted for age
[2]Adjusted for sex
[3]Adjusted for age and sex
*p<0.05; **p<0.01; ***p<0.001

For peer review only

The association of sociodemographic characteristics with self-perceived access to information and adherence to Covid-19 preventive measures among the migrant origin population is presented in Table 4. Longer length of residence and excellent Finnish/Swedish skills were associated with perceived sufficient information on Covid-19. Women were generally more likely to adhere to preventive measures than men. Older age was associated with a lower likelihood of reporting staying at home with flu symptoms and downloading the Koronavilkku contact tracing app, while tertiary education was associated with a greater likelihood of downloading the Koronavilkku app. Secondary education was associated with being less likely, whereas excellent Finnish/Swedish skills were associated with being more likely to avoid shaking hands with others. Those who have lived in Finland for 7-12 years were less likely to use a face mask than those who have lived in Finland for a shorter period.

[Table 4 about here]

The association of self-perceived access to information with adherence to preventive measures is presented in Table 5. When the migrant origin population was examined as one group, the association was statistically significant for keeping a safety distance to others only. When examined by region of origin, statistically significant differences emerged in the population born in Africa in case of all preventive measures except for downloading the Koronavilkku app. Because of widened conficence intervals in the subgroup analyses, interaction analyses were run to examine whether the associations for this group really differed from other regional groups. The p-values for the differences of Africa group vs. other groups combined were p=0.101 for staying at home with flu symptoms; p=0.018 for following improved hand and coughing hygiene; p=0.074 for using a face mask; p=0.255 for keeping a safety distance to others; p=0.034 for avoiding shaking hands with others; and p=0.504 for downloading the Koronavilkku app. Based on these, it appears that the association of perceived access to information and following preventive measures was different for this regional group.

[Table 5 about here]

For peer review only

Table 4 The association of sociodemographic characteristics with perceived adequate access to information, adherence to Covid-19 related preventive measures, and Covid-19 infection among migrant origin persons

	Perceived access to information	Staying home with flu symptoms	Improved hand and coughing hygiene	Use of face mask	Keeping a safety distance to others	Avoiding shaking hands with others	Downloaded Koronavilkku app
	OR (95%CI)	OR (95%CI)	OR (95%CI)	OR (95%CI)	OR (95%CI)	OR (95%CI)	OR (95%CI)
**Sex[1]**							
Men	1.00	1.00	1.00	1.00	1.00	1.00	1.00
Women	1.22 (0.79–1.89)	1.07 (0.60–1.93)	**2.10 (1.33–3.31)****	**1.70 (1.23–2.33)****	**2.12 (1.26–3.56)****	**2.41 (1.48–3.92)*****	1.08 (0.84–1.39)
**Age[2]**							
21-34 years	1.00	1.00	1.00	1.00	1.00	1.00	1.00
35-49 years	0.94 (0.57–1.56)	**0.25 (0.14–0.47)*****	1.01 (0.58–1.76)	1.10 (0.76–1.59)	1.36 (0.73–2.52)	1.06 (0.62–1.82)	**0.61 (0.46–0.82)****
50-66 years	1.39 (0.73–2.66)	**0.24 (0.12–0.48)*****	0.93 (0.51–1.69)	1.30 (0.86–1.97)	2.09 (0.99–4.40)	1.29 (0.68–2.43)	**0.35 (0.25–0.49)*****
**Education[3]**							
Basic education or less	1.00	1.00	1.00	1.00	1.00	1.00	1.00
Secondary education	0.99 (0.50–1.94)	0.58 (0.14–2.40)	0.57 (0.22–1.46)	0.73 (0.42–1.25)	0.87 (0.36–2.10)	**0.38 (0.15–0.95)***	0.94 (0.63–1.41)
Tertiary education or higher	0.66 (0.34–1.27)	0.82 (0.19–3.56)	1.25 (0.49–3.20)	1.08 (0.62–1.88)	0.86 (0.36–2.07)	1.09 (0.42–2.86)	**1.72 (1.15–2.58)****
**Economic activity[3]**							
Unemployed, student or other	1.00	1.00	1.00	1.00	1.00	1.00	1.00
Employed full-time/part-time	0.69 (0.43–1.13)	1.18 (0.63–2.24)	1.21 (0.76–1.94)	1.11 (0.79–1.56)	1.58 (0.87–2.87)	0.87 (0.54–1.38)	0.92 (0.71–1.20)
**Length of residence in Finland[3]**							
3-6.99 years	1.00	1.00	1.00	1.00	1.00	1.00	1.00
7-11.99 years	1.15 (0.63–2.11)	0.93 (0.30–2.84)	0.50 (0.23–1.09)	**0.45 (0.27–0.75)****	1.21 (0.52–2.82)	0.68 (0.34–1.38)	0.92 (0.71–1.20)
12+ years	**1.94 (1.05–3.57)***	0.85 (0.31–2.30)	0.50 (0.25–1.00)	0.62 (0.38–1.01)	1.35 (0.64–2.84)	1.05 (0.56–1.97)	0.92 (0.71–1.20)
**Finnish/Swedish proficiency[3]**							
Intermediate/beginner or less	1.00	1.00	1.00	1.00	1.00	1.00	1.00
Excellent	**2.71 (1.62–4.53)*****	0.98 (0.51–1.86)	0.83 (0.52–1.30)	1.04 (0.76–1.44)	1.28 (0.72–2.26)	**1.73 (1.06–2.84)***	1.13 (0.87–1.47)

[1]Adjusted for age
[2]Adjusted for sex
[3]Adjusted for age and sex
*p<0.05; **p<0.01; ***p<0.001

For peer review only

Table 5 The association of adequate perceived access to information with following preventive measures by region of origin[1]

	Staying home with flu symptoms	Following improved hand and coughing hygiene	Using a face mask	Keeping a safety distance to others	Avoiding shaking hands with others	Downloading the Koronavilkku app
	OR (95%CI)	OR (95%CI)	OR (95%CI)	OR (95%CI)	OR (95%CI)	OR (95%CI)
General population	0.58 (0.08–4.31)	0.72 (0.23–2.24)	0.95 (0.46–1.97)	0.99 (0.27–3.59)	1.20 (0.16–9.00)	1.78 (0.90–3.52)
Born abroad total	1.58 (0.60–4.16)	0.73 (0.37–1.43)	1.49 (0.88–2.53)	**2.54 (1.15–5.61)***	1.53 (0.71–3.33)	1.03 (0.63–1.69)
Russia or former Soviet Union	1.01 (0.27–3.74)	1.25 (0.46–3.39)	2.87 (1.35–6.11)	2.51 (0.80–7.90)	1.57 (0.46–5.37)	2.05 (0.87–4.81)
Estonia	3.19 (0.44–23.28)	0.25 (0.04–1.77)	0.46 (0.08–2.62)	4.56 (0.56–37.26)	1.01 (0.15–6.95)	1.18 (0.22–6.37)
Rest of Europe, North America and Oceania	2.86 (0.46–17.83)	0.74 (0.12–4.43)	1.94 (0.57–6.65)	4.22 (0.87–20.40)	**6.36 (1.42–28.51)***	1.38 (0.49–3.85)
Middle East and North Africa	0.54 (0.09–3.41)	1.46 (0.15–14.60)	1.98 (0.56–6.97)	0.61 (0.10–3.77)	0.87 (0.17–4.44)	1.39 (0.45–4.29)
Rest of Africa	**8.29 (1.76–39.01)****	**15.06 (1.19–190.10)***	**6.13 (1.73–21.76)****	**6.63 (1.70–25.86)****	**15.50 (3.44–69.76)*****	0.55 (0.13–2.27)
Asia	0.40 (0.04–3.93)	0.40 (0.09–1.82)	1.56 (0.36–6.71)	2.49 (0.30–20.43)	0.13 (0.01–1.18)	1.08 (0.37–3.14)

[1]Adjusted for age and sex; the reference group are those who percieved they did not receive fully adequate information on COVID-19 and related restrictive measures.

*p<0.05; **p<0.01; ***p<0.001

For peer review only

For peer review only - http://bmjopen.bmj.com/site/about/guidelines.xhtml

DISCUSSION

Perceived access to information and self-reported adherence to preventive measures were high both in the migrant origin and the general population. In the general population, higher education, whereas in the migrant origin population, longer length of residence and excellent Finnish/Swedish language skills, were associated with higher perceived access to information. In the general population, being a woman, older age, and higher education were positively associated with adherence to preventive measures. While women reported higher adherence to preventive measures also in the migrant origin population, the association of other sociodemographic characteristics was less consistent than in the general population. Perceived sufficient access to information was associated with keeping a safety distance to others in the migrant origin population. In the analyses by region of origin, perceived access to information was associated with adherence to all preventive measures except for downloading the Koronavilkku contact tracing app among persons born in Africa.

Despite overall high perceived access to information, migrant origin populations reported they would have needed more information on Covid-19 more frequently than the general population. Furthermore, some regional group differences were observed, which may be attributable to the observed lower Finnish/Swedish language proficiency and shorter length of residence in some groups. Findings on the association of proficiency in Finnish/Swedish with perceived access to information are consistent with findings of a qualitative study conducted among Russian, Arabic, and Somali-speaking populations at the start of the Covid-19 pandemic in Finland[8]. In the current study, perceived access to information was poorer in those who spoke Finnish/Swedish at intermediate level. This may reflect the complexity of crisis communications. Those who do not have sufficient Finnish/Swedish skills, and for whom official information on the Finnish context was not available in own language may be at a particular risk of not receiving sufficient information. While majority (91%) of migrant origin persons reported using Finnish media, use of international media (85%) and social media (82%), which may not necessarily convey accurate information of relevance to the Finnish context, were also frequently reported[16]. Future studies should explore the association of used sources with perceived access to information.

The observed association between length of residence with perceived access to information may be related to higher likelihood of attaining better Finnish/Swedish language proficiency with longer length of residence in Finland. It may also reflect accumulation of better general knowledge on where to source relevant information. The observed association of higher education with higher perceived access to information among the general population in this study is consistent with previous studies conducted among general populations[24]. Persons with higher education may be more skilled in seeking health-related information and may have better health literacy[25].

Self-reported adherence to preventive measures was overall high both among the general and migrant origin population in the current study, which is consistent with findings from Norway[16] and Switzerland[26], but contradictory with findings from France[27]. Partially contradictory findings may be attributable to the fact that the study in France focused specifically on a disadvantaged migrant origin group from one specific region of origin, whereas the other studies included more heterogenous populations. In the current study, some differences in adherence to preventive measures were observed by region of origin. Lower adherence to preventive measures among persons born in Russia/FSU and Estonia compared with other groups may partially reflect behavioural patterns and official recommendations in the country of origin of participants. In Russia and Estonia, adherence to preventive measures has been

For peer review only - http://bmjopen.bmj.com/site/about/guidelines.xhtml

reportedly influenced by the authorities' ambivalent and dismissive attitudes and the citizens' perceived lack of support[28-29]. In contrast, persons born in the Middle East and North Africa and the rest of Africa reported very high adherence to all preventive measures in this study, except for downloading the contact tracing app. It is possible that these groups overreported adherence to preventive measures due to social desirability. However, persons originating from the Middle East and Africa also had the highest incidence of Covid-19 in Finland[30], therefore it is possible that these groups were more motivated to follow recommendations due to higher perceived risk of Covid-19 infection.

Women reported adherence to preventive measures more often than men both in the general and migrant origin populations, which is in consistency with previous studies[24]. Also in other areas of health and health-related behaviours, women generally have healthier habits than men[18, 31]. Findings of a positive association of older age with improved hand and coughing hygiene, use of face mask, and keeping a safety distance to others in the general population is also in consistency with previous studies[24, 32]. In contrast, among the migrant origin population, older age was associated with being less likely to stay at home with flu symptoms. The reasons for this should be explored in future studies. Working conditions may also be associated with adherence to preventive measures. Less than a third of participants in the MigCOVID Survey who were working or training reported they were able to work remotely, 72% were able to take care of their hand hygiene at work, 56% were able to keep a safety distance to others[16]. Future studies should also explore the association of working conditions, such as ability to work remotely and avoid close contact with others, as well as the association of job security, with adherence to preventive measures.

Migrant origin populations reported downloading the contact tracing app less frequently than the general population, and the inverse association of older age with downloading the app. This is in consistency previous studies and has been suggested to be related to lower digital competency among older adults[33-34]. Some of the participants indicated distrust towards authorities as a reason for not downloading the app, which is a concern raised among migrant origin populations also in previous studies[35]. Higher education was associated with higher likelihood of downloading the contact tracing app both among the general and migrant origin populations. The observed differences in how sociodemographic characteristics are associated with adherence to preventive measures in migrant origin compared with the general population point to a need for tailoring population-level measures for them to be effective in influencing behaviours of diverse population groups.

In the current study, little association between perceived access to information and adherence to preventive measures was observed. Both perceived access to information and self-reported adherence to preventive measures were high, which may explain lack of the observed associations. However, when examined by region of origin, the association of adequate access to information and adherence to preventive measures was particularly observed among persons born in Africa. A large proportion of persons belonging to this group were of Somali origin, which is a very close-knit community. Over the course of Covid-19, Somali origin community representatives and non-governmental associations were very active in distributing information within the community. Also the Somali-speaking TV channel was active in disseminating information on Covid-19. Other possible reasons for the observed association should be explored in further studies. During the Covid-19 pandemic in Finland, social listening was introduced to gather signals from diverse population groups on their perceptions and needs related to the crisis[36]. Multilingual crisis communications were developed in close collaboration with regional health and welfare professionals, key community representatives and migrant origin populations. In addition to these, Covid-19 incidence was regularly followed and the MigCOVID Survey, which constitutes the basis for this study,

For peer review only - http://bmjopen.bmj.com/site/about/guidelines.xhtml

was launched to gather population-based data on experiences during Covid-19 among migrant origin populations. When further developing future crisis preparedness, it may be worthwhile to consider use of additional systematic theory-driven tools, such as the framework for evaluating the effectiveness and sustainability of behaviourally and culturally informed interventions in complex settings, recently launched by the World Health Organisation[37].

Significant strengths of the current study are its population-based study design, representative sample, and availability of the general population reference group. The survey was available in 18 of the most commonly spoken languages in Finland, reducing language barriers for majority of the participants. Participation barriers were further reduced through provision of the questionnaire in both electronic and paper-based format, as well as by providing an alternative to respond via a telephone interview. Some limitations also need to be addressed. Self-reported access to information does not provide information on the quality of the used sources or whether the information was understood and applied as intended. There may be some report bias in the self-reported adherence to preventive measures due to social desirability. It may also be that respondents reported adherence to preventive measures, even though they may have not adhered to them in all situations. Measured access to information, that may have reduced potential response bias was not included in the current survey due to restrictions in the length of the questionnaire. However, when possible, researches designing similar surveys in the future are encouraged to consider also including measured access to information.

When conducting surveys in multiple languages, there is a risk that the consistency of the items across the language versions may be jeopardized and that the translation process may alter the meaning of the survey items. In the current study, the translations were made by professional interpreters and have been reviewed by persons speaking the language in question, cross-checking them with the original questionnaire. Additionally, the multilingual interviewers, who were trained in the methodology of conducting surveys, importance of standardization, and why the different questions were being asked, have also reviewed the questionnaires both from the perspective of consistency and conservation of the intended meaning. While it is still possible that some issues remained unnoticed, it is anticipated that these measures considerably reduced the problem of systematic misinterpretations related to translation error or the intended meaning of the items.

Considerable measures were taken to increase participation rate, however the 18 languages in which the questionnaire was available did not exhaustively cover all of the different languages spoken by the participants. Those who did not participate may have had poorer literacy skills, poorer command of Finnish/Swedish, and may have been likely to speak the languages in which the survey was provided. The languages selected in the survey covered the most commonly spoken languages in Finland. These broadly speaking (but not exactly) corresponded to the languages in which multilingual communications were produced in Finland. Therefore, it is possible that those who did not participate may have been at a greater risk of not receiving sufficient information. Furthermore, those who did not participate may have been less interested in their health and hence less likely to seek information or adhere to preventive measures. Non-response bias was addressed in this study through application of inverse probability weights[23]. These were based on register information on sociodemographics, and for the participants in the FinMonik sample, also earlier survey data on socioeconomic position and type of municipality of residence[4]. While the sample size and participation rate were substantial compared with other surveys conducted among migrant origin population during Covid-19, not all of the analyses could have been performed by region of origin.

For peer review only - http://bmjopen.bmj.com/site/about/guidelines.xhtml

In conclusion, this study provided evidence on importance of multilingual communications in securing equity in access to information among migrant origin populations in crisis situations. While policy and practice acknowledging the increasing cultural and linguistic diversity of populations has been evidently lacking prior to the pandemic, a number of countries have developed measures to improve access to up-to-date crisis communications in multiple languages during Covid-19. These measures need to be carefully documented and synthecised to identify best practices and to encourage mutual learning, as well as to facilitate better preparedness in future crises. Population diversity is a global trend that is accelerating rapidly. Therefore, it is imperative that multilingual and crisis communications are included in crisis preparedness plans. Countries must be ready to produce crisis communications in multiple languages without a significant time lag from the onset of a crisis to ensure adequate access to information among diverse population groups. Findings on differences in sociodemographic characteristics associated with access to information and adherence to preventive measures across migrant origin populations and also in comparison with the general population highlight the need for acknowledging not only population's linguistic, but also cultural diversity. It is imperative to apply comprehensive theory-driven monitoring and impact assessment measures to analyse the needs, perceptions, and attitudes of diverse population groups during crisis situations to gain insights on drivers of behaviours, as well as to identify most effective strategies for influencing behaviors.

For peer review only

Author contributions: NS conceptualised the paper. NS drafted the manuscript with assistance of TP. TP conducted the literature review. EL conducted the statistical analyses. PK and AC have provided their critical comments for developing the manuscript. All authors have participated in interpreting the results and reviewing drafts. All authors have read and approved the manuscript sent for publication.

Funding: The MigCOVID Survey and the FinHealth 2017 follow-up Study were funded from the Finnish parliament's additional budget allocated for COVID-19 research (grant number N/A). NS and EL received funding from the Coping of disabled persons and persons of migrant origin in exceptional and crisis situations – Building the future based on experiences during Covid-19 (Building the Future) project supported by the European Social Fund (grant number S22389). TP was funded by the Impact of coronavirus epidemic on wellbeing among foreign born population (MigCOVID) Survey. The funding bodies had no role in study design; in the collection, analysis and interpretation of data; in the writing of the report; or in the decision to submit the article for publication.

Acknowledgements: The authors would like to acknowledge all of the participants in the MigCOVID Survey and the Health 2017 Study, fieldwork personnel, as well as the experts involved in planning and data management of the studies.

Data availability statement: Data are available upon reasonable request.

Competing interest statement: There are no competing interests for any author.

Ethics approval: All participants were made aware of the voluntary nature of participation in the surveys and that by participating, they permitted the use of their personal information according to the data protection notification on handling personal data. The MigCOVID Survey received ethical permission from the Ethical Committee of THL (THL/4061/6.02.01/2020). The FinHealth 2017 follow-up Study received ethical permission from the Ethics Committee II of the Helsinki and Uusimaa hospital region (HUS/2391/2020).

For peer review only

For peer review only - http://bmjopen.bmj.com/site/about/guidelines.xhtml

1 Diaz E, Norredam M, Aradhya S, et al. Situational brief: Migration and COVID-19 in Scandinavian countries, *Econ Geogr* 2021.

2 Li SL, Pereira RHM, Prete CA, et al. Higher risk of death from COVID-19 in low-income and non-White populations of Sao Paulo, Brazil, *BMJ Glob Health* 2021;6:10.1136/bmjgh-004959 doi:e004959 [pii] [published Online First: April 01].

3 Hayward SE, Deal A, Cheng C, et al. Clinical outcomes and risk factors for COVID-19 among migrant populations in high-income countries: A systematic review, *J Migr Health* 2021;3:100041 doi:10.1016/j.jmh.2021.100041 [doi].

4 Skogberg N, Koponen P, Lilja E, et al. Impact of Covid-19 on the health and wellbeing of persons who migrated to Finland: The MigCOVID Survey 2020-2021, 2021.

5 Schmidt H, Wild E, Schreyögg J. Explaining variation in health information seeking behaviour – Insights from a multilingual survey, *Health policy (Amsterdam)* 2021;125:618-26 doi:10.1016/j.healthpol.2021.01.008 [published Online First: May].

6 Massaro M, Tamburro P, La Torre M, et al. Non-pharmaceutical Interventions and the Infodemic on Twitter: Lessons Learned from Italy during the Covid-19 Pandemic, *J Med Syst* 2021;45:50-7 doi:10.1007/s10916-021-01726-7 [doi] [published Online First: March 06].

7 Brønholt RLL, Langer Primdahl N, Jensen AMB, et al. "I Just Want Some Clear Answers": Challenges and Tactics Adopted by Migrants in Denmark When Accessing Health Risk Information about COVID-19, *Int J Environ Res Public Health* 2021;18:8932 doi:10.3390/ijerph18178932 [published Online First: Aug 25,].

8 Finell E, Tiilikainen M, Jasinskaja-Lahti I, et al. Lived Experience Related to the COVID-19 Pandemic among Arabic-, Russian- and Somali-Speaking Migrants in Finland, *Int J Environ Res Public Health* 2021;18:10.3390/ijerph18052601 doi:2601 [pii] [published Online First: March 05].

9 Knights F, Carter J, Deal A, et al. Impact of COVID-19 on migrants' access to primary care and implications for vaccine roll-out: a national qualitative study, *Br J Gen Pract* 2021;71:e583-95 doi:10.3399/BJGP.2021.0028 [doi] [published Online First: July 29].

10 Deal A, Hayward SE, Huda M, et al. Strategies and action points to ensure equitable uptake of COVID-19 vaccinations: A national qualitative interview study to explore the views of undocumented migrants, asylum seekers, and refugees, *J Migr Health* 2021;4:100050 doi:10.1016/j.jmh.2021.100050 [doi].

11 Wang C, Tian Q, Zhao P, et al. Disease knowledge and attitudes during the COVID-19 epidemic among international migrants in China: a national cross-sectional study, *Int J Biol Sci* 2020;16:2895-905 doi:10.7150/ijbs.47075 [doi] [published Online First: September 04].

12 Valeriani G, Vukovic IS, Mollica R. Unconventional Answers to Unprecedented Challenges: The Swedish Experience During the COVID-19 Outbreak, *J Prev Med Public Health* 2020;53:233-5.

13 Nezafat Maldonado BM, Collins J, Blundell HJ, et al. Engaging the vulnerable: A rapid review of public health communication aimed at migrants during the COVID-19 pandemic in Europe, *J Migr Health* 2020;1:100004 doi:10.1016/j.jmh.2020.100004 [doi].

14 Skogberg N, Hussein I, Castaneda A. Impact of Covid-19 human mobility restrictions on migrant origin population in Finland, *in book: COVID-19 and Migration: Understanding Pandemic and Human Mobility.Published* 2020;11.

15 Statistics Finland. Immigrants in the population. Official statistics of Finland, 2022. Accessed February 1st, 2023 from: https://www.stat.fi/tup/maahanmuutto/maahanmuuttajat-vaestossa_en.html.

For peer review only - http://bmjopen.bmj.com/site/about/guidelines.xhtml

16 Skogberg N, Koponen P, Lilja E, et al. Access to information, preventive measures and working conditions during the coronavirus epidemic: Findings of the population-based MigCOVID Survey among persons who have migrated to Finland, 2021.

17 Madar AA, Benavente P, Czapka E, et al. COVID-19: information access, trust and adherence to health advice among migrants in Norway, *Archives of public health = Archives belges de santé publique* 2022;80:15 doi:10.1186/s13690-021-00764-4 [published Online First: Jan 4,].

18 Kuusio H, Seppänen A, Jokela S, et al. Ulkomaalaistaustaisten terveys ja hyvinvointi Suomessa: FinMonik-tutkimus 2018-2019, 2020.

19 Borodulin K, Sääksjärvi K. FinHealth 2017 Study: Methods, 2019. Finnish Institute for health and Welfare, THL; Helsinki.

20 United Nations. Standard country or area codes for statistical use (M49). 2021.

21 Graubard BI, Korn EL. Predictive margins with survey data, *Biometrics* 1999;55:652-9.

22 Lehtonen R, Pahkinen E. Practical methods for design and analysis of complex surveys: John Wiley & Sons 2004.

23 Robins JM, Rotnitzky A, Zhao LP. Estimation of regression coefficients when some regressors are not always observed, *J Am Stat Assoc* 1994;89:846-66.

24 Varghese NE, Sabat I, Neumann-Böhme S, et al. Risk communication during COVID-19: A descriptive study on familiarity with, adherence to and trust in the WHO preventive measures, *PloS one* 2021;16:e0250872.

25 Demirci Ş, Uğurluoğlu Ö, Konca M, et al. Socio-demographic characteristics affect health information seeking on the Internet in Turkey, *Health Info Libr J* 2021;38:304-12.

26 Nivette A, Ribeaud D, Murray A, et al. Non-compliance with COVID-19-related public health measures among young adults in Switzerland: Insights from a longitudinal cohort study, *Social science & medicine (1982)* 2021;268:113370 doi:10.1016/j.socscimed.2020.113370 [published Online First: Jan].

27 Gosselin A, Melchior M, Carillon S, et al. Deterioration of mental health and insufficient Covid-19 information among disadvantaged immigrants in the greater Paris area, *J Psychosom Res* 2021;146:110504 doi:S0022-3999(21)00149-5 [pii] [published Online First: July 01].

28 Margraf J, Brailovskaia J, Schneider S. Adherence to behavioral Covid-19 mitigation measures strongly predicts mortality, *PloS one* 2021;16:e0249392 doi:10.1371/journal.pone.0249392.

29 Margraf J, Brailovskaia J, Schneider S. Behavioral measures to fight COVID-19: An 8-country study of perceived usefulness, adherence and their predictors, *Plos one* 2020;15:e0243523.

30 Holmberg V, Salmi H, Kattainen S, et al. Association between first language and SARS-CoV-2 infection rates, hospitalization, intensive care admissions and death in Finland: a population-based observational cohort study, *Clin Microbiol Infect* 2022;28:107-13 doi:10.1016/j.cmi.2021.08.022 [published Online First: Jan].

31 Koponen P, Borodulin K, Lundqvist A, et al. Terveys, toimintakyky ja hyvinvointi Suomessa: FinTerveys 2017 -tutkimus. Terveyden ja hyvinvoinnin laitos, raportti 4/2018.

32 Bearth A, Luchsinger L, Siegrist M. Reactions of older Swiss adults to the COVID-19 pandemic: A longitudinal survey on the acceptance of and adherence to public health measures, *Soc Sci Med* 2021;280:114039.

For peer review only - http://bmjopen.bmj.com/site/about/guidelines.xhtml

33 Dowthwaite L, Fischer J, Vallejos EP, et al. Public Adoption of and Trust in the NHS COVID-19 Contact Tracing App in the United Kingdom: Quantitative Online Survey Study, *J Med Internet Res* 2021;23:e29085.

34 Skarpa PE, Garoufallou E. Information seeking behavior and COVID-19 pandemic: A snapshot of young, middle aged and senior individuals in Greece, *Int J Med Inf* 2021;150:104465.

35 Abba-Aji M, Stuckler D, Galea S, et al. Ethnic/racial minorities' and migrants' access to COVID-19 vaccines: A systematic review of barriers and facilitators. *J Migr Health* 2022:100086.

36 Lohiniva A, Sibenberg K, Austero S, et al. Social Listening to Enhance Access to Appropriate Pandemic Information Among Culturally Diverse Populations: Case Study From Finland *JMIR Infodemiology* 2022;2(2):e38343 doi: 10.2196/38343

37 World Health Organization. Guide to evaluating behaviourally and culturally informed health interventions in complex settings. 2022. https://www.who.int/europe/publications/i/item/WHO-EURO-2022-6045-45810-65956

For peer review only

For peer review only - http://bmjopen.bmj.com/site/about/guidelines.xhtml

STROBE 2007 (v4) Statement—Checklist of items that should be included in reports of *cross-sectional studies*

Section/Topic	Item #	Recommendation	Reported on page #
**Title and abstract**	1	(*a*) Indicate the study's design with a commonly used term in the title or the abstract	1,2
		(*b*) Provide in the abstract an informative and balanced summary of what was done and what was found	2
**Introduction**			
Background/rationale	2	Explain the scientific background and rationale for the investigation being reported	4
Objectives	3	State specific objectives, including any prespecified hypotheses	4
**Methods**			
Study design	4	Present key elements of study design early in the paper	5
Setting	5	Describe the setting, locations, and relevant dates, including periods of recruitment, exposure, follow-up, and data collection	5
Participants	6	(*a*) Give the eligibility criteria, and the sources and methods of selection of participants	5
Variables	7	Clearly define all outcomes, exposures, predictors, potential confounders, and effect modifiers. Give diagnostic criteria, if applicable	5,6
Data sources/ measurement	8*	For each variable of interest, give sources of data and details of methods of assessment (measurement). Describe comparability of assessment methods if there is more than one group	5,6
Bias	9	Describe any efforts to address potential sources of bias	*6*
Study size	10	Explain how the study size was arrived at	5
Quantitative variables	11	Explain how quantitative variables were handled in the analyses. If applicable, describe which groupings were chosen and why	5,6
Statistical methods	12	(*a*) Describe all statistical methods, including those used to control for confounding	6
		(*b*) Describe any methods used to examine subgroups and interactions	6
		(*c*) Explain how missing data were addressed	6
		(*d*) If applicable, describe analytical methods taking account of sampling strategy	6
		(*e*) Describe any sensitivity analyses	-
**Results**			

For peer review only

Participants	13*	(a) Report numbers of individuals at each stage of study—eg numbers potentially eligible, examined for eligibility, confirmed eligible, included in the study, completing follow-up, and analysed	5
		(b) Give reasons for non-participation at each stage	5
		(c) Consider use of a flow diagram	-
Descriptive data	14*	(a) Give characteristics of study participants (eg demographic, clinical, social) and information on exposures and potential confounders	16
		(b) Indicate number of participants with missing data for each variable of interest	16-19
Outcome data	15*	Report numbers of outcome events or summary measures	16-19
Main results	16	(*a*) Give unadjusted estimates and, if applicable, confounder-adjusted estimates and their precision (eg, 95% confidence interval). Make clear which confounders were adjusted for and why they were included	16-19
		(*b*) Report category boundaries when continuous variables were categorized	-
		(*c*) If relevant, consider translating estimates of relative risk into absolute risk for a meaningful time period	-
Other analyses	17	Report other analyses done—eg analyses of subgroups and interactions, and sensitivity analyses	8
**Discussion**			
Key results	18	Summarise key results with reference to study objectives	8
Limitations	19	Discuss limitations of the study, taking into account sources of potential bias or imprecision. Discuss both direction and magnitude of any potential bias	9
Interpretation	20	Give a cautious overall interpretation of results considering objectives, limitations, multiplicity of analyses, results from similar studies, and other relevant evidence	8,9
Generalisability	21	Discuss the generalisability (external validity) of the study results	10
**Other information**			
Funding	22	Give the source of funding and the role of the funders for the present study and, if applicable, for the original study on which the present article is based	11

*Give information separately for cases and controls in case-control studies and, if applicable, for exposed and unexposed groups in cohort and cross-sectional studies.

Note: An Explanation and Elaboration article discusses each checklist item and gives methodological background and published examples of transparent reporting. The STROBE checklist is best used in conjunction with this article (freely available on the Web sites of PLoS Medicine at http://www.plosmedicine.org/, Annals of Internal Medicine at http://www.annals.org/, and Epidemiology at http://www.epidem.com/). Information on the STROBE Initiative is available at www.strobe-statement.org.

For peer review only
